# Conserved transcriptional connectivity of regulatory T cells in the tumor microenvironment informs new combination cancer therapy strategies

**DOI:** 10.1038/s41590-023-01504-2

**Published:** 2023-05-01

**Authors:** Ariella Glasner, Samuel A. Rose, Roshan Sharma, Herman Gudjonson, Tinyi Chu, Jesse A. Green, Sham Rampersaud, Izabella K. Valdez, Emma S. Andretta, Bahawar S. Dhillon, Michail Schizas, Stanislav Dikiy, Alejandra Mendoza, Wei Hu, Zhong-Min Wang, Ojasvi Chaudhary, Tianhao Xu, Linas Mazutis, Gabrielle Rizzuto, Alvaro Quintanal-Villalonga, Parvathy Manoj, Elisa de Stanchina, Charles M. Rudin, Dana Pe’er, Alexander Y. Rudensky

**Affiliations:** 1 Immunology Program, Sloan Kettering Institute, Memorial Sloan Kettering Cancer Center, New York, NY USA; 2 Program for Computational and Systems Biology, Sloan Kettering Institute, Memorial Sloan Kettering Cancer Center, New York, NY USA; 3 Institute of Biotechnology, Life Sciences Centre, Vilnius University, Vilnius, Lithuania; 4 Human Oncology & Pathogenesis Program, Sloan Kettering Institute, Memorial Sloan Kettering Cancer Center, New York, NY USA; 5 Department of Pathology & Laboratory Medicine, Sloan Kettering Institute, Memorial Sloan Kettering Cancer Center, New York, NY USA; 6 Department of Medicine, Thoracic Oncology Service, New York, NY USA; 7 Antitumor Assessment Core Facility, New York, NY USA; 8 Molecular Pharmacology Program, Sloan Kettering Institute, Memorial Sloan Kettering Cancer Center, New York, NY USA; 9 Howard Hughes Medical Institute, Sloan Kettering Institute, Memorial Sloan Kettering Cancer Center, New York, NY USA

**Keywords:** Tumour immunology, Cancer immunotherapy

## Abstract

While regulatory T (T_reg_) cells are traditionally viewed as professional suppressors of antigen presenting cells and effector T cells in both autoimmunity and cancer, recent findings of distinct T_reg_ cell functions in tissue maintenance suggest that their regulatory purview extends to a wider range of cells and is broader than previously assumed. To elucidate tumoral T_reg_ cell ‘connectivity’ to diverse tumor-supporting accessory cell types, we explored immediate early changes in their single-cell transcriptomes upon punctual T_reg_ cell depletion in experimental lung cancer and injury-induced inflammation. Before any notable T cell activation and inflammation, fibroblasts, endothelial and myeloid cells exhibited pronounced changes in their gene expression in both cancer and injury settings. Factor analysis revealed shared T_reg_ cell-dependent gene programs, foremost, prominent upregulation of VEGF and CCR2 signaling-related genes upon T_reg_ cell deprivation in either setting, as well as in T_reg_ cell-poor versus T_reg_ cell-rich human lung adenocarcinomas. Accordingly, punctual T_reg_ cell depletion combined with short-term VEGF blockade showed markedly improved control of PD-1 blockade-resistant lung adenocarcinoma progression in mice compared to the corresponding monotherapies, highlighting a promising factor-based querying approach to elucidating new rational combination treatments of solid organ cancers.

## Abstract (web summary)

Single-cell and spatial analyses of conserved regulatory T (T_reg_) cell-dependent transcriptional states of diverse accessory cell types in mouse and human lung cancer suggest rational T_reg_ cell targeting-based combination therapy for PD-1 blockade-resistant tumors.

## Main

Diverse stromal cell types found within the tumor microenvironment (TME) can support cancer initiation and progression by acting as accessory cells, yet their relationships and interdependencies remain poorly understood. Cells of the innate and adaptive immune system, when mobilized by immunotherapeutic agents, have been implicated in limiting cancer progression, yet some of the very same cell types can support tumor growth either directly or indirectly by facilitating tumor-promoting functions of other accessory cell types. T_reg_ cells, expressing the transcription factor Foxp3, are highly enriched in human solid organ cancers and their experimental animal models, and at sites of inflammation and injury, where they exert both their essential immunosuppressive function and distinct tissue repair-promoting modalities^1–4^. Depletion of T_reg_ cells results in restraint of tumor growth in numerous experimental cancer models^5–9^. Nevertheless, some tumors eventually progress after an initial response to T_reg_ depletion^5^. The latter can be due to waning functionality of effector T cells due to negative regulation by co-receptors, foremost PD-1, expected to occur primarily in PD-1 blockade-responsive tumors expressing PD-L1. An alternative, yet not mutually exclusive, explanation, is that T_reg_ cell depletion induces compensatory modulation of key accessory cell types in the TME, which may affect predominantly PD-1 nonresponsive cancers. Thus, early changes in diverse cellular components of the TME upon short-term T_reg_ cell depletion may directly and indirectly impact its overall effect on tumor growth. Thus, we sought to elucidate the interplay between T_reg_ cells and other cellular components of the TME by investigating early changes in their features upon T_reg_ depletion in experimental cancer settings. Specifically, we wished to use a genetically engineered mouse model that is characterized by natural evolution of the TME, pronounced T_reg_ cell presence, resistance to PD-1 blockade and close resemblance to human disease. Therefore, we used *Kras*^LSL-G12D/WT^*Trp53*^fl/fl^ mice harboring a *Foxp3*^GFP-DTR^ allele (KP-DTR), in which intratracheal infection with a Cre-expressing replication-deficient adenovirus induces lung adenocarcinoma (LuAd) formation. These mice offer a well-established model of non-small cell lung cancer (NSCLC) in humans, a disease where only some respond to PD-1/PD-L1 blockade-based therapies^7,10–12^. Our studies revealed that T_reg_ cells profoundly affect the transcriptional programs of key accessory cells including endothelial cells, fibroblasts, monocytes and macrophages in the TME. Moreover, these T_reg_ cell dependencies of the transcriptional states of accessory cells are largely conserved in human lung cancer.

## Results

### Early responses of tumor microenvironment cells to T_reg_ cell depletion

**Fig. 1:**
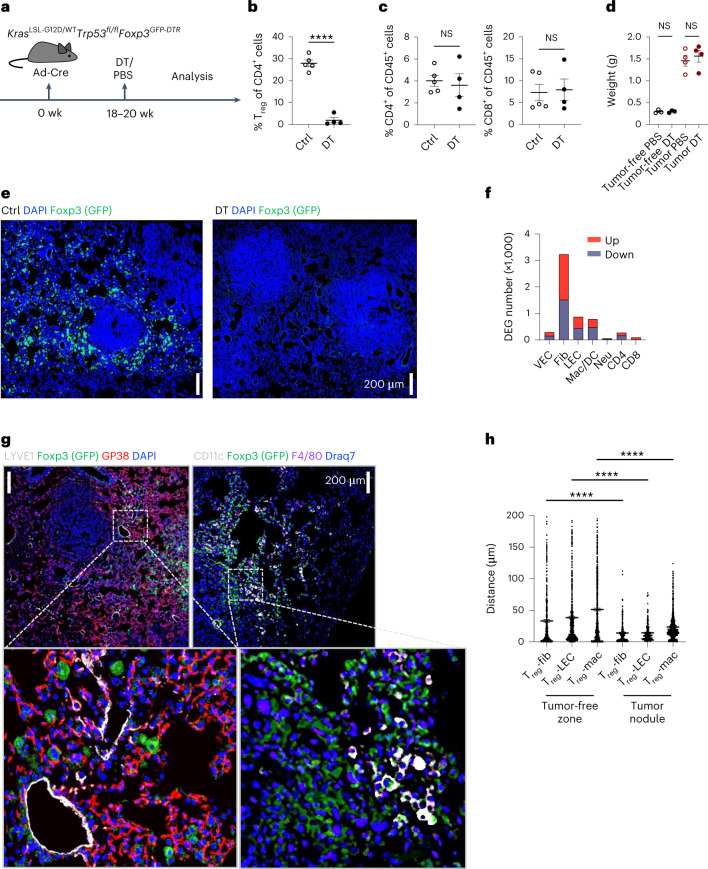
Early transcriptional responses of principal accessory cell populations in the lung adenocarcinoma TME to T_reg_ cell depletion. **a**, Schematic of the experimental design. **b**,**c**, Quantification of T_reg_ (CD4^+^Foxp3^+^) one-tailed unpaired *t*-test *P* = 12.87, d.f. = 7 ****P < 0.0001 and Tcon (TCRβ^+^CD4^+^ and TCRβ^+^CD8^+^) cell populations; left, one-tailed *t*-test *P* = 0.3799, d.f. = 7, not significant (NS) *P* = 0.3576; right, one-tailed *t*-test *P*= 0.1925, d.f. = 7, NS *P* = 0.4264, in tumor-bearing lungs 48 h after diphtheria toxin (DT) or PBS (Ctrl) administration. **d**, Quantification of lung weight in tumor-free and tumor-bearing mice 48 h after DT-induced T_reg_ cell depletion. One-way analysis of variance (ANOVA) followed by Sidak’s multiple-comparisons test. Tumor-free PBS versus tumor-free DT, *P* = 0.004037, d.f. = 10 NS *P* > 0.9999; tumor PBS versus tumor DT, *P* = 0.7450, d.f. = 10, NS *P* = 0.9787. **e**, Representative IF staining of Foxp3^+^ cells in tumor-bearing lungs of Ctrl and DT-treated mice. **f**, Numbers of upregulated (red) or downregulated (blue) DEGs (*P* < 0.05) 48 h after DT or PBS administration identified by bulk RNA-seq analysis of the indicated cell subsets. Fib, fibroblasts; Neu, neutrophils; Mac, macrophages; CD4 and CD8, effector CD4^+^ and CD8^+^ T cells. **g**, Representative IF staining of the indicated cell types. **h**, Quantification of distances between T_reg_ cells and the indicated cell types. One-way ANOVA, alpha = 0.05, followed by Tukey’s multiple-comparison test T_reg_-Fib tumor-free zone versus tumor nodule, *q* = 8.041, d.f. = 2544 ****P < 0.0001. T_reg_-LEC tumor-free zone versus tumor nodule *q* = 10.08, d.f. = 2544, ****P < 0.0001, T_reg_-Mac versus tumor-free zone versus tumor nodule *q* = 17.79, d.f. = 2544, ****P < 0.0001. At least 200 cells were counted in each comparison. Three independent sections per mouse were analyzed. Three and four mice were used in each group in two independent experiments. Data are presented as the mean ± s.e.m. (**b**–**d**) (**b** and **c**) *N* = Ctrl-5, DT-4, (**d**) *N* = 3 tumor-free PBS, 3 tumor-free DT, 4 tumor PBS, 4 tumor DT. Data are presented as the mean ± s.e.m. Source data

**Extended Data Fig. 1:**
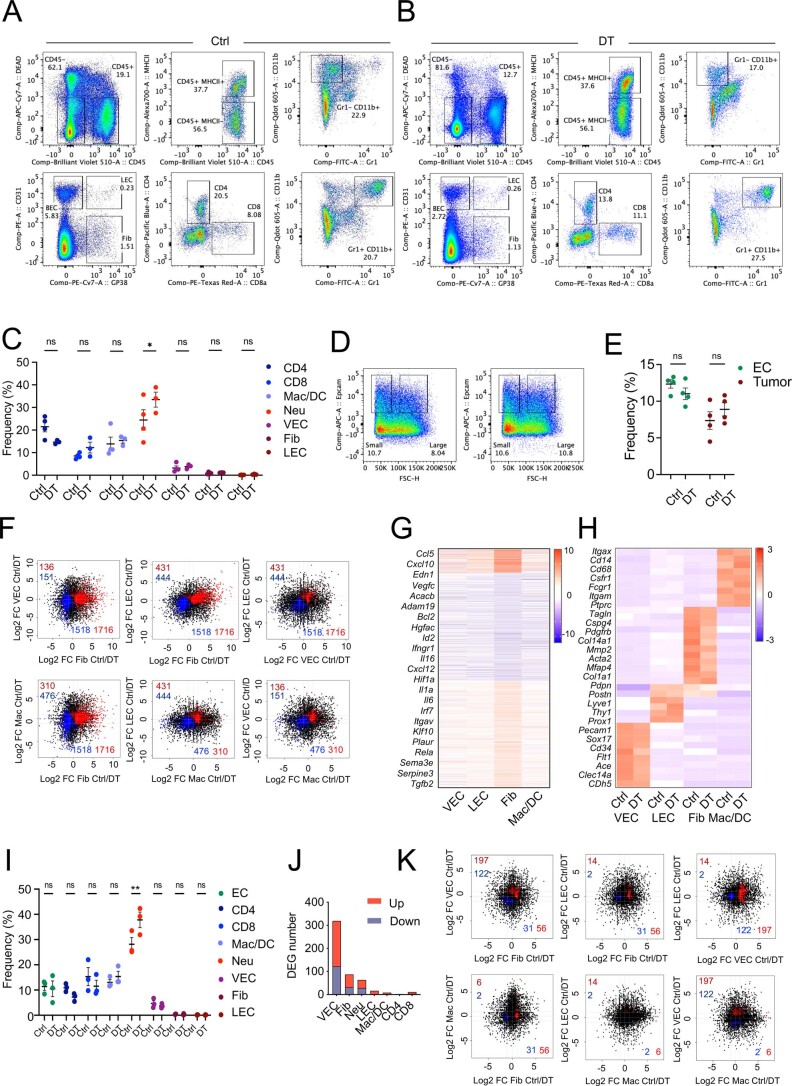
Short-term Treg depletion in lung KP adenocarcinoma bearing mice. (**a**, **b**, **d**) Representative gating of normal and tumor (EpCAM) cells, (D) TCRβ^+^CD4^+^ (CD4), TCRβ^+^CD8^+^ (CD8) T cells, myeloid cells (MHCII^+^GR1^-^CD11b^+^), neutrophils (Neu) (MHCII^-^GR1^+^CD11b^+^), vascular endothelial cells (VEC) (CD45^-^CD31^+^GP38^−^), fibroblasts (Fib) (CD45^-^CD31^-^GP38^+^), and lymphatic endothelial cells (LEC) (CD45^−^CD31^+^GP38^+^) (A, B) in KP-lungs in diphtheria toxin (DT, *N* = 3) and PBS (Ctrl, *N* = 4) mice and (D) in tumor-free lungs (DT, *N* = 4), PBS (Ctrl, *N* = 4). (**c**, **e**) Cell frequencies in KP tumors from (A, B, D). Data represent mean ± SEM of one of two independent experiments. (**c**) Two-way ANOVA alpha = 0.05, Šídák’s multiple comparisons CD4 t = 2.254, df = 35 ns P = 0.1953, CD8 t = 1.235, df = 35 ns P = 0.8320, MHCII+/Gr1- CD11b+ (MAC/DC) t = 0.5098 df = 35 ns P = 0.9987, MHCII-/Gr1+ CD11b+ (Neu) t = 2.985, df = 35, * P = 0.0355, VEC, t = 0.2030, df = 35 ns P>0.9999, Fib t = 0.09821, df = 35 ns P>0.9999, Lec t = 0.08549, df = 35 ns P>0.9999. (**e**) Two-way ANOVA, Alpha = 0.05, Tukey’s multiple comparisons EC, t = 1.056. df = 12, ns P = 0.5261 Tumor t = 1.217, df = 12, ns P = 0.4332. (**f**) Fold change (FC) deferentially expressed genes (DEG). (**g**) k-means clustering of FC DEG between DT and Ctrl. Columns - log2 FC (DT/Ctrl) for cells, each row is a gene. Select genes are labeled. (**h**) Z-score-normalized counts for selected genes in (G). (**i**) Cell frequencies in tumor-free DT and Ctrl lungs. Two-way ANOVA, alpha = 0.05, Šídák’s multiple comparisons. Epcam t = 0.3437, df = 37, ns P>0.9999, CD4 t = 1.413, df = 32 ns 0.769, CD8 t = 1.434, df = 32 ns P = 0.7550, MHCII+/Gr1- CD11b+ (MAC/DC) t = 0.8971, df = 32, ns P = 0.9771, MHCII-/Gr1+ CD11b+ (Neu) t = 3.664, df = 32 ** P = 0.0071, VEC t = 0.3854, df = 32 ns P>0.9999, Fib t = 0.008845, df = 32 ns P>0.9999, LEC t = 0.01251, df = 32 ns P>0.9999. Data represent mean ± SEM of one of two independent experiments *N* = DT-3, PBS-3. (**j**) DEG Numbers. (**k**) FC DEG in cells isolated from tumor-free lungs of DT vs Ctrl mice. (F, J, K) DEG - differentially expressed genes (p<0.05). Red-upregulated, blue-downregulated. Source data

To enable temporally controlled T_reg_ cell depletion in KP adenocarcinomas, we generated *Kras*^LSL-G12D/WT^*Trp53*^fl/fl^*Foxp3*^GFP-DTR^ mice, in which all T_reg_ cells express the diphtheria toxin receptor (DTR)^13^. We reasoned that since T_reg_ cells are typically found in the tumor margins, early compensatory responses of key accessory cell types—tumor-associated fibroblasts, vascular endothelial cells (VECs) and lymphatic endothelial cells (LECs), and macrophages (Mac)—to T_reg_ cell depletion likely precede effects on the tumor growth. Because the expansion of activated self-reactive T cells, observed 72–96 h after DT-mediated T_reg_ cell depletion in *Foxp3*^*GFP-DTR*^ mice, induces pronounced inflammatory responses^13^, we sought to minimize these confounding factors by analyzing early transcriptional responses of KP tumor cells, lung epithelial cells (ECs), VECs, LECs, macrophages and T cells 48 h following DT administration to tumor-bearing KP-DTR mice (Fig. 1a,b and Extended Data Fig. 1a,e). As expected, pronounced local and systemic T cell activation and inflammation, typically elicited by an extended T_reg_ cell depletion regimen, were not observed (Fig. 1c and Supplementary Fig. 1c), and tumor volume was unaffected at this early time point (Fig. 1d) even though neutrophils were moderately increased (Extended Data Fig. 1c,k). Highly efficient tumoral T_reg_ cell depletion in situ was confirmed by immunofluorescence (IF) microscopy of DT-treated as compared to control (Ctrl) mice, in which T_reg_ cells were found mainly at the boundaries of tumor foci (Fig. 1e). Bulk RNA-sequencing (RNA-seq) analyses of cell subsets purified by fluorescence-activated cell sorting (FACS) from the lungs of DT-treated KP adenocarcinoma-bearing KP-DTR mice showed pronounced changes in gene expression in LECs, macrophages and fibroblasts, while T cells, which are considered the main targets of T_reg_ cell suppression, changed the least (Fig. 1f and Supplementary Table 1). Among accessory cells, the most pronounced transcriptional responses were observed in fibroblasts, endothelial cells and CD11c^+^ myeloid cells, highlighting T_reg_ cell ‘connectivity’ to these cell types in tumor-bearing lungs (Extended Data Fig. 1f–h and Supplementary Table 1). Importantly, DT-induced T_reg_ cell ablation in tumor-free control KP-DTR mice resulted in minor, if any, changes in gene expression in all lung cell populations analyzed with the exception of VECs (Extended Data Fig. 1j,k). This was consistent with the predominantly intravascular localization of T_reg_ cells in the lung of unchallenged mice in contrast to their heavy presence in the cancerous lung parenchyma (Extended Data Fig. 1f,g)^14^. These results suggest that the observed transcriptional changes in accessory cells in cancerous lungs are not due to a systemic response to T_reg_ cell depletion. Next, we investigated whether shared groups of genes underwent modulation in different accessory cell types and observed correlated gene expression changes in endothelial cells and fibroblasts (Extended Data Fig. 1f, g). These included programs related to endothelial-to-mesenchymal transition (EndMT)-related genes (*Id2, Itgav* and *Cxcl12*), which were previously shown to be modulated by T_reg_ cells in the hair follicles^15^, and inflammation-related genes (*Il6*, *Ccl5*, *Acacb*, *Ccl22*, *Arg1* and *Tnfrsf18)*, whose expression is affected by T_reg_ cells in adipose tissue in the context of metabolic inflammation and muscle injury^16,17^ (Extended Data Fig. 1g). Cell-type-specific gene expression changes confirmed the shared gene expression changes were not due to sample impurities (Extended Data Fig. 1h). Considering the early transcriptional response of several TME cell types to T_reg_ depletion, we assessed whether T_reg_ cells were found in the proximity of these ‘first responders’ using IF analysis of tumor-bearing lungs. Indeed, GFP-DTR^+^ T_reg_ cells were found in markedly closer proximity to Lyve-1^+^ LECs, GP38^+^ fibroblasts and F4/80^+^ macrophages within and near tumor nodules than in areas further away from tumor nodules in the same tumor-bearing lung (Fig. 1g,h). Collectively, we have shown that T_reg_ cells are highly connected in the KP TME.

### Single-cell analysis of tumoral T_reg_ cell ‘connectivity’

**Fig. 2:**
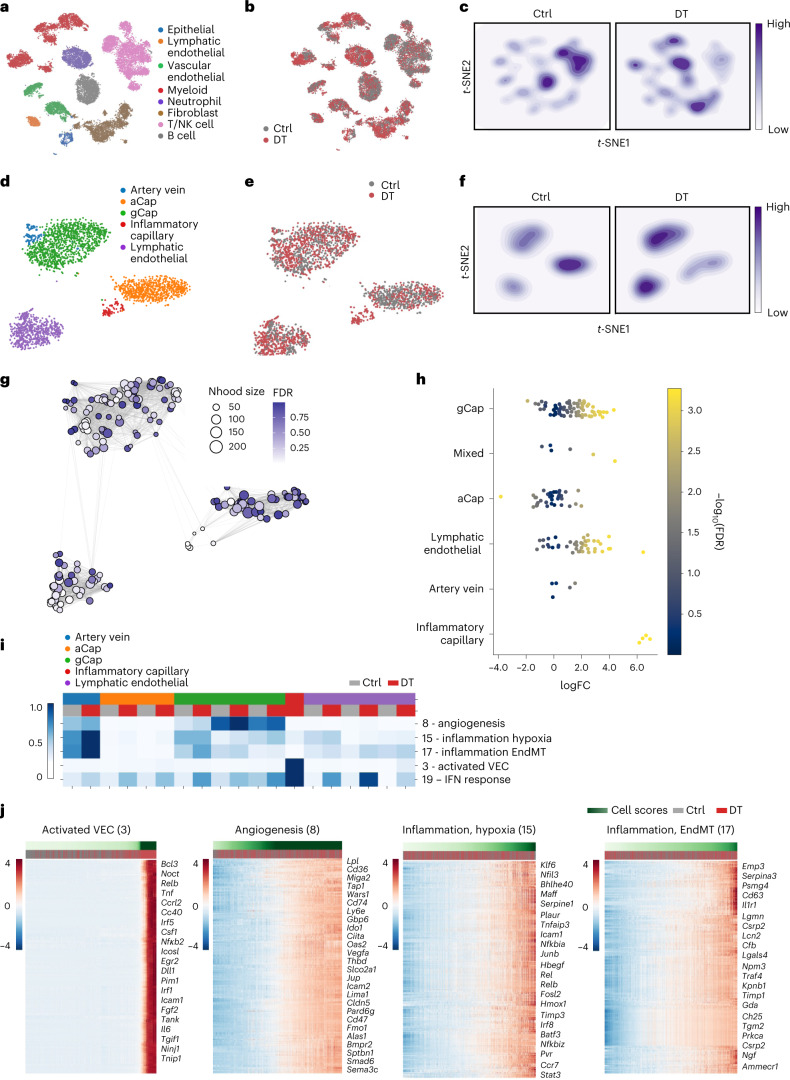
Single-cell transcriptomic analysis of ‘T_reg_ cell dependencies’ of accessory cell states in mouse lung adenocarcinoma tumor microenvironment. **a**,**b**, *t*-distributed stochastic neighbor embedding (*t*-SNE) plots (27,000 cells) representing cell populations from major cell lineages isolated from 48 h DT-treated or PBS-treated (Ctrl) tumor-bearing lungs (three mice per group) colored by cell type (**a**) and condition (**b**). **c**, A density plot showing the distribution of cells between experimental conditions. **d**,**e**, *t*-SNE plots (2,815 cells) representing distribution of the VEC populations colored by subtype (**d**) and condition (**e**). **f**, A density plot of endothelial cells showing the distribution of cells between experimental conditions. **g**, Graph of neighborhoods of endothelial cells computed using MiloR and *t*-SNE embedding. Each dot represents a neighborhood and is color coded by the false discovery rate (FDR)-corrected *P* value (alpha = 1) quantifying the significance of enrichment of DT cells compared to control in each neighborhood. The size of the dot represents the number of cells in the neighborhood. **h**, Swarm plot depicting the log fold change of differential cell-type abundance in DT-treated versus control samples in each neighborhood across different endothelial cell types. Each dot represents a neighborhood and is color coded by the FDR-corrected *P* value (alpha = 1) quantifying the significance of enrichment of DT cells compared to control in each neighborhood. A neighborhood is classified as a cell type if it comprises at least 80% of cells in the neighborhood, otherwise it is called ‘mixed’. **i**, Heat map showing average factor cell score in each cluster for each experimental condition in the VEC population. The scores were row normalized between 0 and 1. Each row represents a factor, and each column represents a cluster in a specific experimental condition. The clusters are grouped based on their phenotype. **j**, Gene expression heat maps showing the top 200 genes that correlated the most with the imputed activated VEC factor indicated (Methods). Each column represents a cell; cells are ordered based on their factor score (in ascending order from left to right) indicated by the green bar. Select genes of interest are noted on the right. **b**, **e**, **h** and **i**; Ctrl, PBS, gray; DT, red. Source data

**Extended Data Fig. 2:**
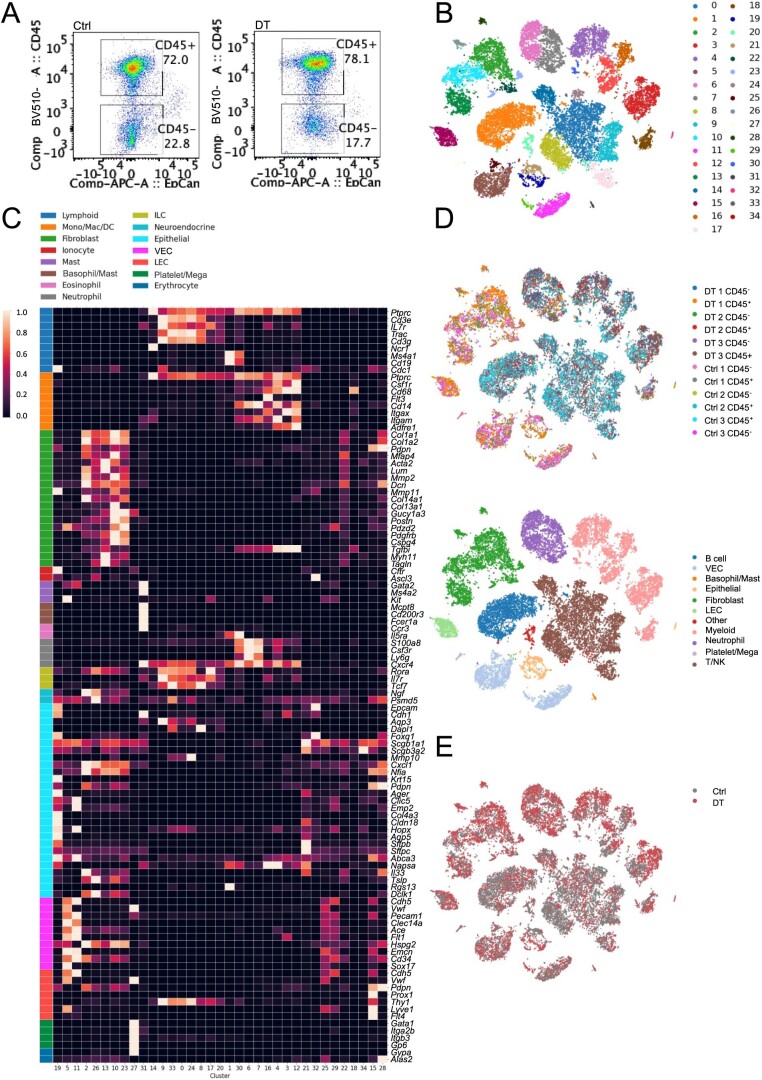
scRNA-seq analysis and annotation of major TME cell types in lung KP adenocarcinomas. (**a**) Sorting strategy. CD45^+^ and CD45^−^ cells were sorted from lungs of PBS (Ctrl) and DT treated (48 hr) mice (3 mice per group) harboring KP lung tumors. (**b**) t-SNE plots embedding (27,606 cells) representing distribution of all the cells isolated in (A), colored by PhenoGraph clusters (k = 30) (left), or sample (right) (related to Fig. 2a, which shows major cell lineages). (**c**) Heatmap showing the average expression of cell type specific markers in each cluster. The rows are genes and columns are clusters. Shown expression is row normalized between 0–1 and genes are grouped to indicate the subtype they typically are associated with. All the genes used for annotation are shown. (**d**) t-SNE embedding (same as B) colored by lineages inferred using the average expression of each cluster shown in the heatmap in (C). (**e**) t-SNE embedding reflecting experimental conditions (Ctrl: PBS, gray; DT: diphtheria toxin, red). Source data

**Extended Data Fig. 3:**
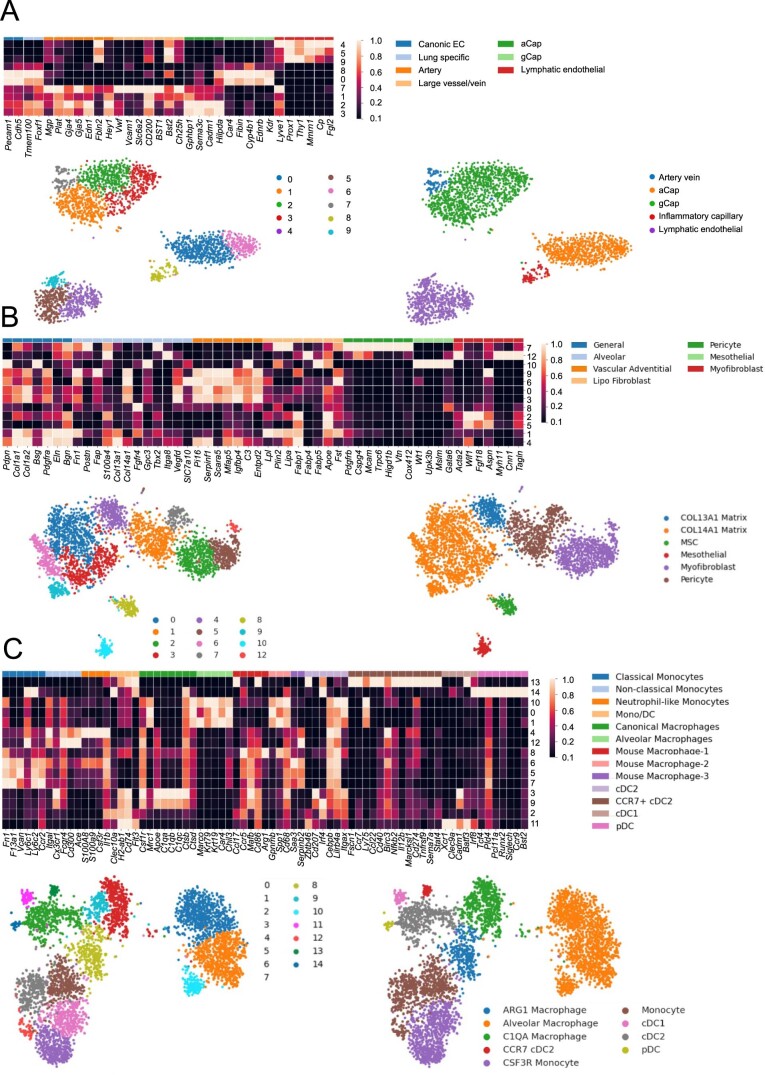
Heatmaps of Treg depletion-induced gene expression changes in fibroblasts, endothelial and myeloid cells in lung KP adenocarcinomas. (**a**) Heatmap showing the average expression of known endothelial markers in each endothelial cell cluster. Rows indicate cluster and columns indicate genes. The heatmap is column normalized between 0–1 and the genes are grouped to indicate the subtype they typically are associated with (top). t-SNE embeddings (2815 cells) (bottom) representing distribution of endothelial cells color coded by their cluster identity inferred using the gene expression pattern for each cluster shown in the heatmap (left) and cell type annotation derived from the heatmap above (right). All genes used for annotation are shown. (**b**) Same as (A) for fibroblasts. (**c**) Same as (A) for myeloid cells. Source data

**Extended Data Fig. 4:**
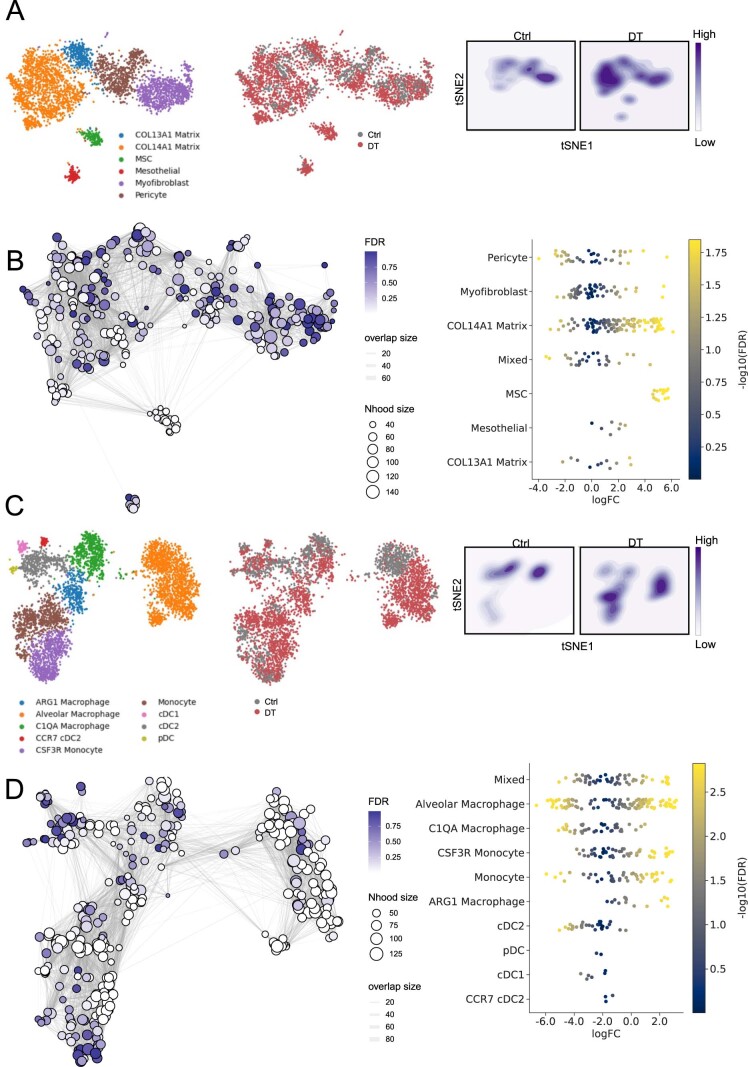
Neighborhood analysis of Treg depletion-induced gene expression changes in endothelial cells, fibroblast and myeloid cells in lung KP adenocarcinomas. (**a**) t-SNE embedding of fibroblasts (3,791 cells) (top) color coded by cell subtype (left) or experimental condition (right). A density plot of the distribution of fibroblasts between conditions (bottom). Ctrl – PBS, gray; DT - diphtheria toxin, red. (**b**) Graph of neighborhoods of fibroblast cells computed using MiloR and embedded on t-SNE (top). Each dot represents a cellular neighborhood and is color coded by the FDR corrected p-value (alpha = 1) quantifying the significance of enrichment of DT cells compared to control in each neighborhood. The size of the dot represents the number of cells in the neighborhood. (bottom). Swarm plot depicting the log-fold change in differential abundance of DT treated cells against control cells in each neighborhood across different fibroblast cell types. Each dot represents a neighborhood and is color coded by the FDR corrected p-value (alpha = 1) quantifying the significance of enrichment of DT cells compared to control in each neighborhood. A neighborhood is classified as a cell type if it comprises at least 80% of cells in the neighborhood, or called ‘mixed’ otherwise. (**c**) Same as (A) for myeloid cells. (**d**) Same as (B) for myeloid cells. Source data

To explore the impact of T_reg_ cells on the diverse cell states in the TME, we performed single-cell RNA sequencing (scRNA-seq) of sorted CD45^−^ and CD45^+^ cell populations using the 10X platform (Extended Data Fig. 2a). These populations were isolated from tumor-bearing lungs of KP-DTR mice treated for 48 h with DT or vehicle control 3 months after adenoviral Cre-driven tumor initiation (Fig. 1a). After pre-processing, we clustered cells using PhenoGraph^18^ and annotated clusters using expression of known markers into major cell types (Extended Data Fig. 2b–d). To ensure our inferences were robust, we focused on the major hematopoietic and non-hematopoietic cell types in the TME that had substantial numbers of cells. The final processed datasets included LECs, VECs, LECs, fibroblasts, lymphoid cells and myeloid cells (macrophages, monocytes, dendritic cells (DCs) and neutrophils; Fig. 2a). Similarly to population-level assessments, scRNA-seq showed that short-term T_reg_ cell depletion had profound effects on transcriptional features of fibroblasts, myeloid and endothelial cells compared to lymphocytes (Extended Data Fig. 2e and Fig. 2a–c). To gain deeper insight into the phenotypic response of accessory cells whose transcriptomes were most affected by T_reg_ removal—endothelial cells, fibroblasts and myeloid cells, we separately clustered and embedded each subtype to ascribe finer-grain identities (Fig. 2d and Extended Data Fig. 3; for annotation strategy see Methods). Furthermore, we used Milo^19^ to quantify changes in abundance of subpopulations and cell states after T_reg_ cell depletion (Methods). We found several cell states affected by T_reg_ cell depletion, with the most pronounced phenotypic shifts in capillary VECs, mesenchymal stem cells (MSCs), *Col14a1* matrix fibroblasts, monocytes and macrophages (Fig. 2d–h and Extended Data Fig. 4a–d). Therefore, T_reg_ cell depletion markedly affected the distribution and abundancies of several cell states and subsets in the TME.

### Shared and distinct T_reg_ cell-dependent gene programs

We then sought to characterize genes that respond to T_reg_ cell depletion in these key accessory cell subsets. We used factor analysis to characterize gene expression programs—sets of genes whose expression changes in a coordinated way in a specific set of cells and assessed their differential usage in cell populations from control or DT mice to elucidate the response to T_reg_ cell depletion. Specifically, factor analysis methods are well suited to decompose data into factors, which represent coordinated expression programs across cells and reduce the impact of noise on analysis, which can be dominant at an individual gene level^20^. We used single-cell hierarchical Poisson factorization (scHPF), designed specifically for scRNA-seq^21,22^ and applied it to each cell lineage separately to dissect the observed gene expression changes. Each cell and gene present in the expression matrix was assigned a score for each factor, enabling biological interpretation of that factor (see Supplementary Table 2 for factor gene and cell matrices). Factors were robust to random initializations of the model and robust to slight changes in parameters (Methods and Supplementary Fig. 1).

We reasoned that gene programs most affected by T_reg_ cell presence would have differential factor cell scores between the control and DT conditions. To evaluate this systematically, we computed the average cell score of every factor in each cluster for each condition (Fig. 2i) and identified those that have higher averages in DT compared to control. In the endothelial lineage, we identified four major gene programs that were robust to random initializations (Supplementary Fig. 1), were biologically relevant and had significantly differential cell scores (Mann–Whitney test; Methods) following T_reg_ cell depletion compared to control in at least one of the endothelial cell subtypes (Fig. 2i). We then visualized expression of the genes with the highest factor loadings in the relevant cell subtype (Fig. 2j). We observed several notable patterns, including the inflammatory or activated capillary VEC factor (factor 3), a highly T_reg_ cell-dependent factor characterized by cytokine/chemokine-, Notch and nuclear factor-κB (NF-κB) signaling-, and co-stimulation pathway-related gene expression (Fig. 2j; see Supplementary Table 3 for endothelial factors of interest). Other highlighted factors enriched following T_reg_ cell depletion in the endothelial cell population included genes related to the NF-κB signaling pathway (*Nfkbia*, *Rel*, *Hbegf*), inflammation/hypoxia (*Klf6*, *Serpine1*, *Plaur*; factor 15) and vascularization (*Vegfa*, *Thbd* and *Slco2a1*; factor 8), and genes linked to transforming growth factor-beta-induced EndMT (*Emp3*, *Timp1* and *Tgm2*; factor 17). Besides cancer, the latter process is induced in aberrant tissue remodeling and fibrosis^23,24^. These observations indicate that T_reg_ cells impact specific features of certain endothelial cell subsets in the TME.

**Extended Data Fig. 5:**
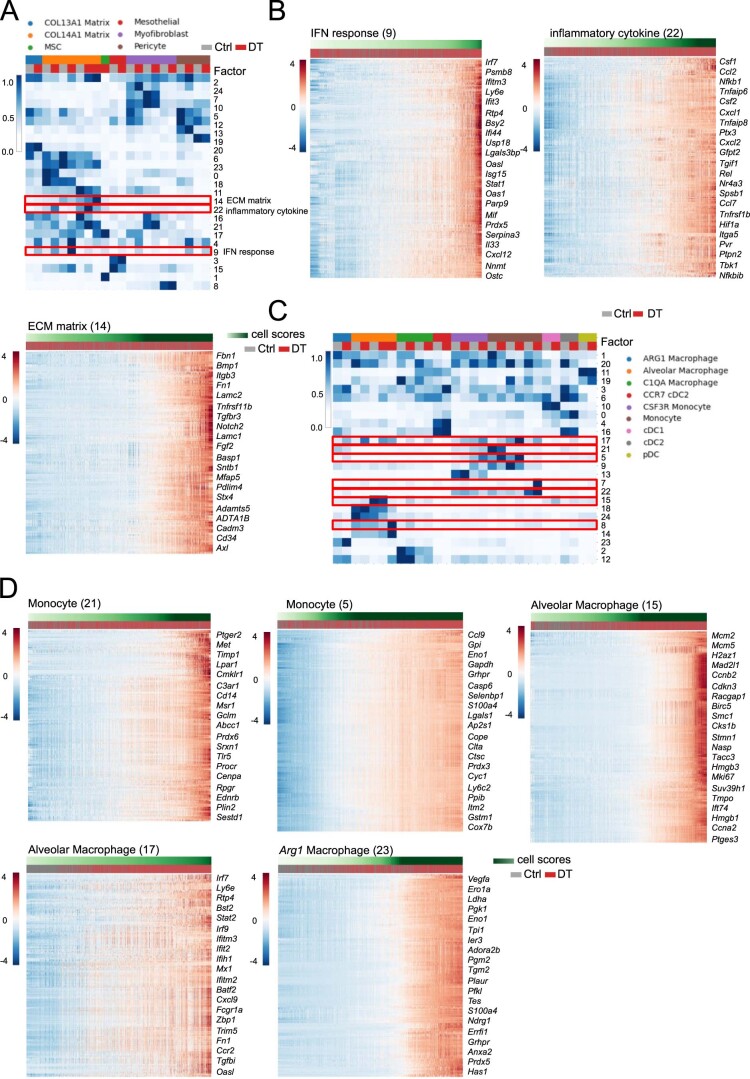
Factor analysis of Treg-dependent gene expression by fibroblasts and myeloid cells in lung KP adenocarcinomas. (**a**) Heatmap showing factor cell score across experimental conditions averaged over each fibroblast cluster in each experimental condition. The rows are factors and columns are clusters for each experimental condition. The clusters are grouped based on the cell type they are associated with. The heatmap is row normalized from 0–1. (**b**) Heatmaps showing the top 200 genes that correlate the most with imputed cell scores of the indicated factors (see Methods) for fibroblast subsets. Each column is a cell; cells are ordered based on their factor score in ascending order from left to right indicated by the green bar. The experimental condition for each cell is indicated by the grey for PBS (Ctrl) and red for diphtheria toxin-treated conditions (DT) bar. Select examples of genes of interest are noted. (**c**) Heatmap as in A for myeloid cells. (**d**) Heatmaps as in B for myeloid cells. Source data

Notably, the observed transcriptomic perturbations were not unique to endothelial cells. The T_reg_ cell depletion-induced gene programs related to interferon (IFN) response, inflammatory cytokines (ICs) and chemokines, STAT3 and interleukin (IL)-6 signaling appeared to be shared across accessory cell populations. The three most differentially expressed gene (DEG) programs observed in fibroblasts following T_reg_ cell depletion included an inflammatory secretory phenotype (*Ccl2*, *Hif1a*, *Rel*, *Cxcl1*; factor 22), IFN response (*Irf7*, *Ifit3*, *Isg15*; factor 9) and ECM-related genes (*Fbn1*, *Fn1*, *Lamc2*, *Notch2*; factor 14; Extended Data Fig. 5a,b and Supplementary Table 4). On the other hand, several factors in monocytes (factors 2, 5, 7, 13, 17, 21 and 22) and macrophages (factors 15, 17 and 23) including IFN and hypoxia response emerged as differentially abundant (Extended Data Fig. 5c,d and Supplementary Table 5; for all significant factors across cell subsets, see Supplementary Table 6). These results suggested that T_reg_ cell communication with various cells in the TME imparted both shared and distinct transcriptional features across and within specific cell populations in either a direct or indirect manner.

### T_reg_ cell dependency of accessory cell states in lung injury

**Fig. 3:**
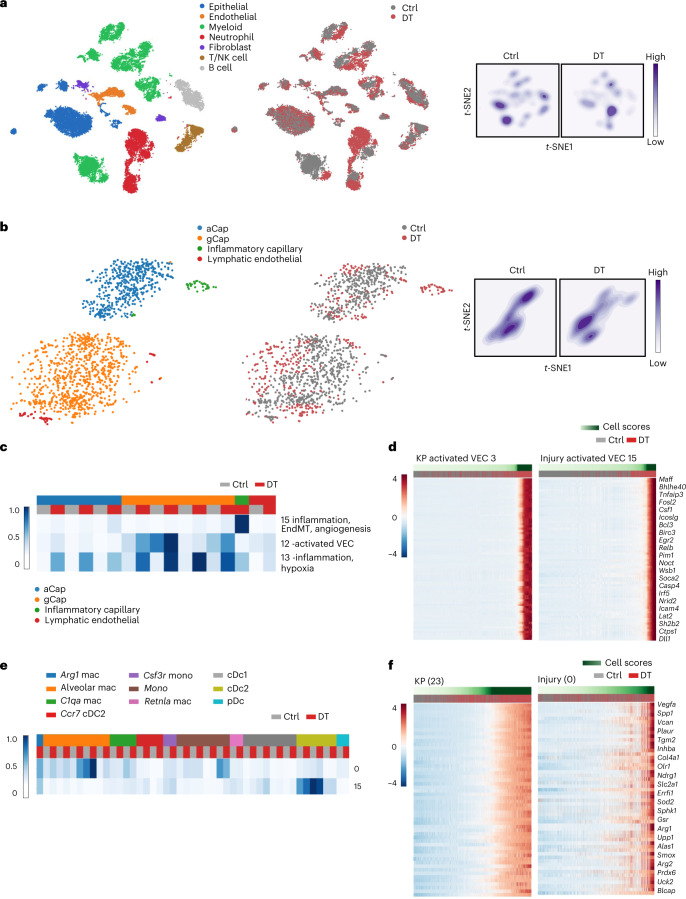
Shared early transcriptional responses induced upon T_reg_ cell depletion in mouse lung adenocarcinoma TME and bleomycin-induced lung inflammation. **a**, *t*-SNE plots (24,592 cells) representing cell populations isolated from the lungs of mice administered with diphtheria toxin (DT) or PBS (Ctrl) for 48 h. Lung injury-induced inflammation was induced in both groups of mice upon bleomycin treatment 21 d before DT/PBS administration. The data represent analysis of three mice per group colored by cell type (left) and condition (middle), and a density of the distribution of cells between conditions (right). **b**, *t*-SNE embedding of endothelial cells isolated from Ctrl and DT after bleomycin administration color coded by cell type (left) or experimental condition (middle), and density plots of the distribution of endothelial cells between conditions (right). **c**, Heat map showing average factor cell score in each cell type for each experimental condition for endothelial cell subsets. The scores were row normalized between 0 and 1. Each row represents a factor, and each column represents an endothelial cell subset in a specific experimental condition. Factors of interest are highlighted by a red box. **d**, Heat map showing the 72 shared genes specific to activated VEC factor in both lung challenge models (Methods and Supplementary Table 9). Each column represents a cell; cells are ordered based on their factor score (in ascending order from left to right), indicated by the green bar. **e**, Heat map showing factor cell score across experimental conditions averaged over each myeloid cluster in each experimental condition for bleomycin-administered cells. The rows are factors and columns are clusters for each experimental condition. The clusters are grouped based on the cell type they are associated with. The heat map shows row-normalized scores from 0 to 1. The left color bar shows the average factor cell score. **f**, Heat maps showing the 54 shared genes between mouse lung tumor and injury-induced inflammation in the *Arg1*^+^ macrophage factor (tumor factor 23 corresponding to injury-induced inflammation factor 0; Supplementary Table 10). Each column is a cell; cells are ordered based on their factor score (in ascending order from left to right) indicated by the green bar. The treatment condition for each cell is indicated by gray for PBS and red for DT bars. Select genes of interest are shown. Source data

**Extended Data Fig. 6:**
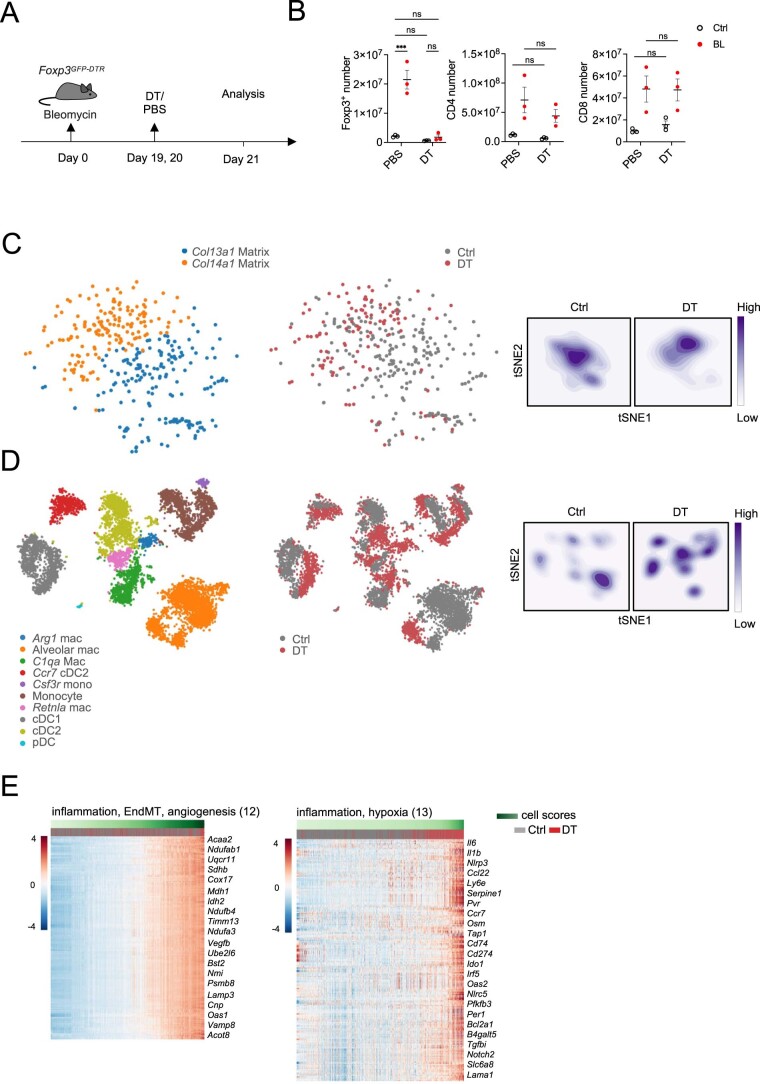
Treg-dependent gene expression changes in endothelial and myeloid cells in bleomycin-induced lung inflammation vs KP adenocarcinomas. (**a**) Schematic of the experimental design. (**b**) Numbers of Treg and effector T cells in Ctrl (PBS) and DT (Diphtheria Toxin) treated lungs, at day 21 after bleomycin administration. (Left) Two-way ANOVA, Alpha = 0.05, followed by Tukey’s multiple comparisons test was performed. PBS Ctrl vs. PBS BL, q = 11.66 df = 8 ***P = 0.0002, PBS Ctrl vs. DT Ctrl q = 0.9285, DF = 8, ns P = 0.9103. PBS Ctrl vs. DT BL q = 0.1986, df = 8 ns P = 0.9989. DT Ctrl vs. DT BL q = 0.7299 df = 8, ns P = 0.9529. Center Two-way ANOVA, Alpha = 0.05, followed by Šídák’s multiple comparisons test was performed. PBS Ctrl vs DT Ctrl t = 0.3479 df = 8 ns P = 0.9997, PBS BL vs DT BL t = 1.575 df = 8 ns P = 0.633. (Right) Two-way ANOVA, Alpha = 0.05, followed by Tukey’s multiple comparisons test was performed. PBS Ctrl Vs DT Ctrl q = 00.7223 df = 8 ns P = 0.9542 PBS BL vs DT BL t = 0.1102 df = 8 ns P = 0.9998. A representative of two independent experiments with 3 mice per group in each is shown. (**c**, **d**) t-SNE embedding of endothelial cells isolated from lungs of DT treated and Ctrl mice color coded by cell type (left) or experimental condition (middle) and density plots of the distribution of endothelial cells between conditions (right). (**c**) fibroblast, (D) myeloid cells. (**e**) Heatmaps showing the top 200 genes that correlate the most with imputed cell scores of the indicated factors for endothelial cells. Each column is a cell; cells are ordered based on their factor score in ascending order from left to right indicated by the green bar. The treatment condition for each cell is indicated by grey (Ctrl) and red (DT) bars. Select genes of interest are shown. Source data

To test whether the T_reg_ cell ‘connectivity’ to key accessory cell types observed in lung cancer represents a generalizable facet of tissue organization, we examined perturbations of their transcriptional states upon identical short-term T_reg_ cell depletion in a setting of bleomycin-induced fibrotic lung inflammation using scRNA-seq analysis (Fig. 3a,b and Extended Data Fig. 6a–d). Not only were all cell populations detected in tumor-bearing lungs also present in inflamed lungs, T_reg_ depletion in this setting also generated similar transcriptional responses (Fig. 3a,b and Extended Data Fig. 6c,d). Independent analysis of the gene programs in the inflamed lung using scHPF (see Supplementary Table 7 for factor matrices) identified T_reg_ cell depletion-associated endothelial factors (Fig. 3c and Supplementary Table 8). We correlated gene scores associated with each factor from lung tumors to lung injury to identify similarities. We found that the activated VEC factor in the lung injury (factor 15) correlated strongly (Pearson correlation > 0.70) with its counterpart in the tumor setting (factor 3), indicating that the same set of genes responded to the loss of T_reg_ cells in both challenges. In fact, 72 of the top 200 genes associated with factor 3 specific to the tumor endothelial cell inflammatory capillary subset were shared with the top 200 genes associated with factor 15 specific to the same subset of cells in the injury model (Fig. 3d and Supplementary Table 9). Other endothelial cell factors, namely inflammation/hypoxia (factor 13), NF-κB signaling and EndMT (factor 12), that were observed in the inflamed lung upon short-term T_reg_ cell depletion also correlated positively, even if weakly, with related tumor factors 15 and 8, respectively (Extended Data Fig. 6e). Consistently, factor analyses of other lineages revealed overlapping differential gene programs between tumor and injury models, including T_reg_ cell depletion-induced gene programs in Arg1^+^ macrophages (Fig. 3e,f and Supplementary Table 10) and IC signatures in Col14a1 matrix fibroblasts. These findings suggested that T_reg_ cell-dependent transcriptional programs are not limited to the TME and can be shared across pathological conditions.

### Spatial distribution of T_reg_ cell-dependent tumor microenvironment gene programs

**Fig. 4:**
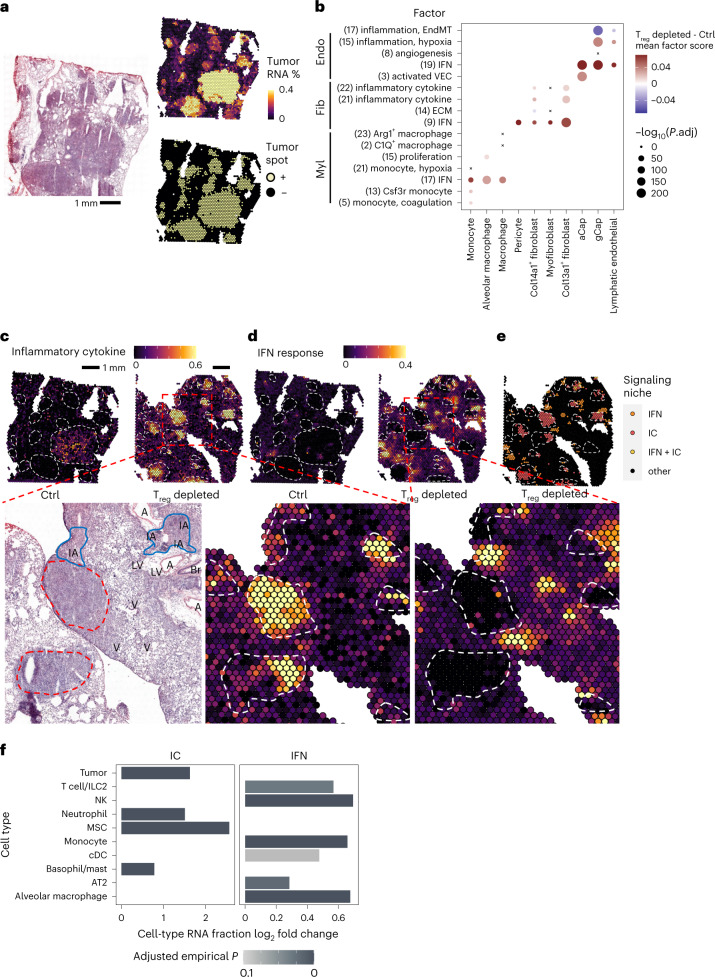
Spatial transcriptomics identifies distinct inflammatory cytokine and IFN signaling niches in lung adenocarcinoma following T_reg_ cell depletion. **a**, Tumor region identification in KP LuAd sections using Visium ST. The fraction of tumor cell RNA in each Visium spot (top right) was determined by BayesPrism deconvolution, binarized (bottom right; Methods), and compared to histological H&E images (left). **b**, Factor scores and Bonferroni-adjusted two-sided *t*-test *P* values differentially expressed factors between control and T_reg_ cell-depleted conditions in ST. **c**,**d**, Representative tissue sections from control (left) or T_reg_ cell-depleted (right) conditions. Tumor regions are outlined, and spots are colored by factor score. Scores represent IC (**c**; 18 genes) or IFN (**d**; 103 genes) gene programs shared across all lineages (Br, bronchi; A/V, artery/vein; LV, lymphatic vessel; IAs, immune cell aggregates). **e**, ST analysis revealed distinct signaling niches. Spots were assigned to niches based on thresholding a gamma distribution fitted to IC or IFN signaling module scores across all spots (Methods). **f**, Enrichment of cell-type RNA fractions in signaling niches. Adjusted empirical *P* value corresponds to the probability of obtaining the mean observed RNA fraction for that cell type (Methods). Fractions with adjusted *P* > 0.01 are not shown. In **a** and **c**–**e**, images are representative of, and analysis performed on (**b** and **f**), one of two serial sections for each of four samples (DT and Ctrl, two biological replicates each). Source data

**Extended Data Fig. 7:**
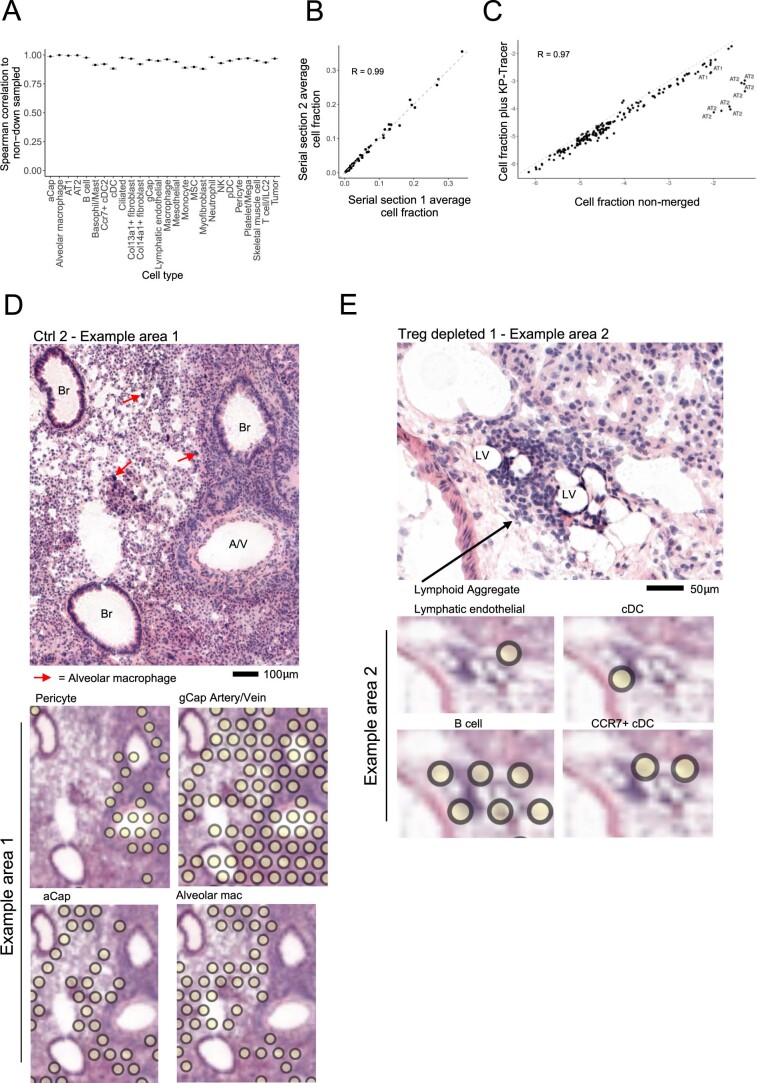
Robustness and validation of BayesPrism deconvolution. (**a**) For each cell type, Spearman’s correlation of cell fraction across all spots was calculated between deconvolution using all available reads and 1 of 20 separate deconvolutions using the available reads downsampled to 90%. Points represent the mean of the 20 Spearman’s correlation calculations and error bars are the minimum and maximum correlation values. (**b**) Comparison of cell fractions across separately deconvolved serial sections. For all four biological samples, the average cell fraction for each cell type is plotted in the first serial section relative to the second. Trend line indicates a slope of 1. Spearman’s correlation is shown. (**c**) Comparison of average log cell fractions in each of 8 tissue sections using the standard scRNA-seq reference or the reference with tumor RNA substituted for KP-Tracer tumor cells. Trend line indicates a slope of 1. Spearman’s correlation is shown. (**d, e**). Examples of positive spots for certain populations of interest are associated with histological features. Images are from representative areas of control and Treg depleted tissue sections. Plots with positive spots display the same example areas in the top of each panel arrangement with the H&E stained image at lower resolution. (Br = bronchi; A/V = artery / vein; LV = lymphatic vessel). Analysis performed on (A-C) and images are representative of (D-E), one of two serial sections for each of four samples (DT and Ctrl two biological replicates each). One experiment was performed. Source data

To gain insights into the spatial organization of the identified accessory cell populations, gene programs and their relationship to transcriptional states of tumor cells, we profiled four tissue sections (two control, two T_reg_ cell depleted) using the 10X Visium platform. We used BayesPrism^25,26^, a Bayesian framework that jointly estimates cell-type fractions and cell-type-specific gene expression using a labeled scRNA-seq reference, to deconvolve each spatial transcriptomics (ST) spot into constituent cell populations. Deconvolution was performed using our scRNA-seq datasets labeled with 26 distinct cell populations selected to optimize granularity, robustness and concordance with underlying histological features in paired H&E-stained sections (Methods, Fig. 4a, Extended Data Fig. 7a–e and Supplementary Table 11). Next, we assessed whether the gene factors that changed upon T_reg_ cell depletion in scRNA-seq were also identified by ST analysis. Consistently, we observed upregulation of endothelial and fibroblast IC and IFN signaling-related gene signatures after T_reg_ cell depletion within spots assigned to the corresponding cell type (Fig. 4b). We also observed increased use of genes associated with the activated VEC factor in capillary aerocyte (aCap) endothelium assigned spots, as well as increased IFN and proliferation related gene signatures in myeloid spots. IC and IFN factors shared many genes across all three analyzed accessory lineages (18 for IC, 103 for IFN), which suggested that similar gene programs were induced across colocalized cell types by common stimuli, indicative of a signaling niche. The spatial behavior of shared genes in these two programs showed localization to two distinct signaling niches in the tissue, with the IC gene program (*Cxcl2*, *Ier3*, *Fosl1*, *Il6*) localized to the tumor core and the IFN response gene program (*Ifit1*, *Stat1*, *Isg15*, *Irf7*) localized to the periphery of, or distal to tumor lesions. Inspection of the same H&E-stained sections confirmed dense tumor cell presence with potential hypoxia and neutrophil infiltration at IC foci, and immune cell aggregates at sites with strong IFN response signal (Fig. 4c–e and Supplementary Table 12). Further, ST analysis revealed concordant differential distribution of tumor cells and accessory cell types within these territories with higher frequency of tumor cells, basophils/mast cells, neutrophils and MSCs in IC territories and a high frequency of T cells/type 2 innate lymphoid cells (ILC2s), natural killer (NK) cells, conventional dendritic cells (cDCs), monocytes and alveolar macrophages in IFN territories (Fig. 4f). Taken together, these results point to two primary inflammatory and spatially distinct modes of lung TME response to T_reg_ cell depletion within tumor mass and tumor margin.

### Tumor states associated with response to T_reg_ cell depletion

KP LuAds adopt a range of recurrent transcriptional states with features of differentiated lung ECs, their progenitors or epithelial progenitors from other tissues including the gastrointestinal tract and liver and EMT (epithelial to mesenchymal transition)-associated ones^27–30^. We next sought to identify potential associations between tumor states and the identified TME niches, that is, IC-positive, IFN-positive and cold (negative) ones. We first identified tumor cells within our ECs by calling KRAS p.Gly12Asp mutations. Because optimized dissociative TME single-cell analysis protocols are suboptimal for capturing tumor cells, we identified only 239 tumor cells within our mouse scRNA-seq dataset. To enable robust deconvolution of tumor cell states, we substituted tumor cells from our scRNA-seq dataset with those from a published dataset that had better capture of KP LuAd cells (KP-tracer dataset; *N* = 18,083)^28^. With this updated reference, we performed an additional spot deconvolution to more accurately capture tumor states in the tissue. TME fractions for other cell types remained relatively unchanged between deconvolutions (Extended Data Fig. 7c).

**Fig. 5:**
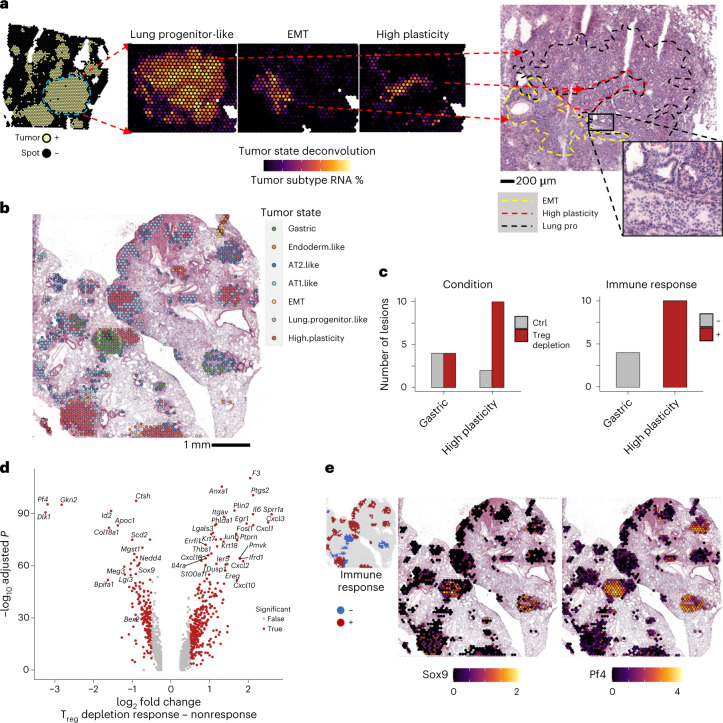
High-plasticity state and heterogeneity revealed by lung adenocarcinoma responses to T_reg_ cell depletion. **a**, ST analysis of tumor states. BayesPrism deconvolution using additional labeled tumor cells from Yang et al.^28^ was performed to assign tumor-state-specific RNA fractions. Correspondence of regions with highlighted differential tumor states (middle) to H&E section is shown (right). Dashed lines denote regions with the indicated dominant tumor states (red, high plasticity; yellow, EMT; black, lung progenitor-like). **b**, Spots labeled by tumor-state cluster. In **a** and **b**, images are representative of, and analysis performed on (**c** and **d**), one of two serial sections for each of four samples (DT and Ctrl, two biological replicates each). **c**, Quantification of tumor lesion area types across T_reg_ cell depletion and control conditions (left) or between tumors with or without detectable immune response in T_reg_ cell-depleted condition (right; *N* = 85 lesion areas). **d**, Differential gene expression (two-sided Wilcoxon test Benjamini–Hochberg adjusted) of tumor spots in lesions with and without immune response to T_reg_ cell depletion. **e**, Log-normalized expression of *Sox9* and *Pf4* (Cxcl4) in a representative tumor-bearing lung section after T_reg_ cell depletion. Inset at top left indicates immune response status of tumor lesion areas. Source data

**Fig. 6:**
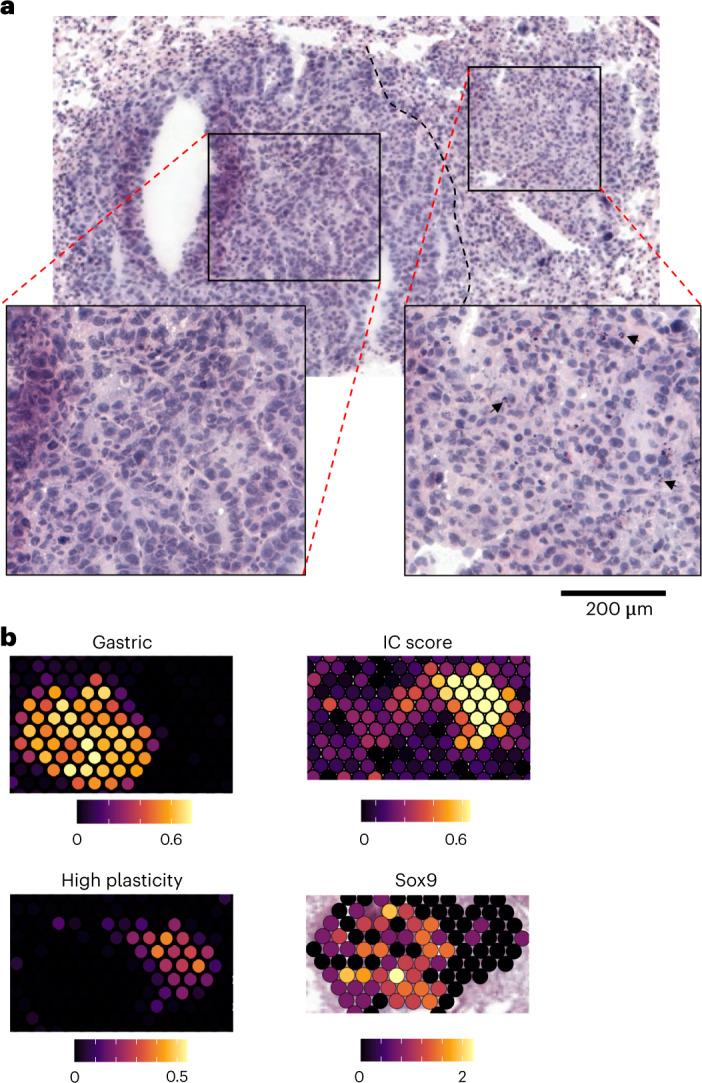
Local histological and immune response heterogeneity following T_reg_ cell depletion. **a**, H&E staining of representative tumor section characterized by histological and immune response state heterogeneity after T_reg_ cell depletion. Insets at bottom represent a zoomed-in view of gastric (left) and high-plasticity (right) areas. Black arrows highlight neutrophil infiltration in a high-plasticity area. **b**, Tumor RNA fraction within highlighted high-plasticity and gastric epithelial states (left) and gene expression modules (right) of tumor lesion shown in **a**. Images are representative of one of two serial sections for each of four samples (DT and Ctrl, two biological replicates each). Source data

**Extended Data Fig. 8:**
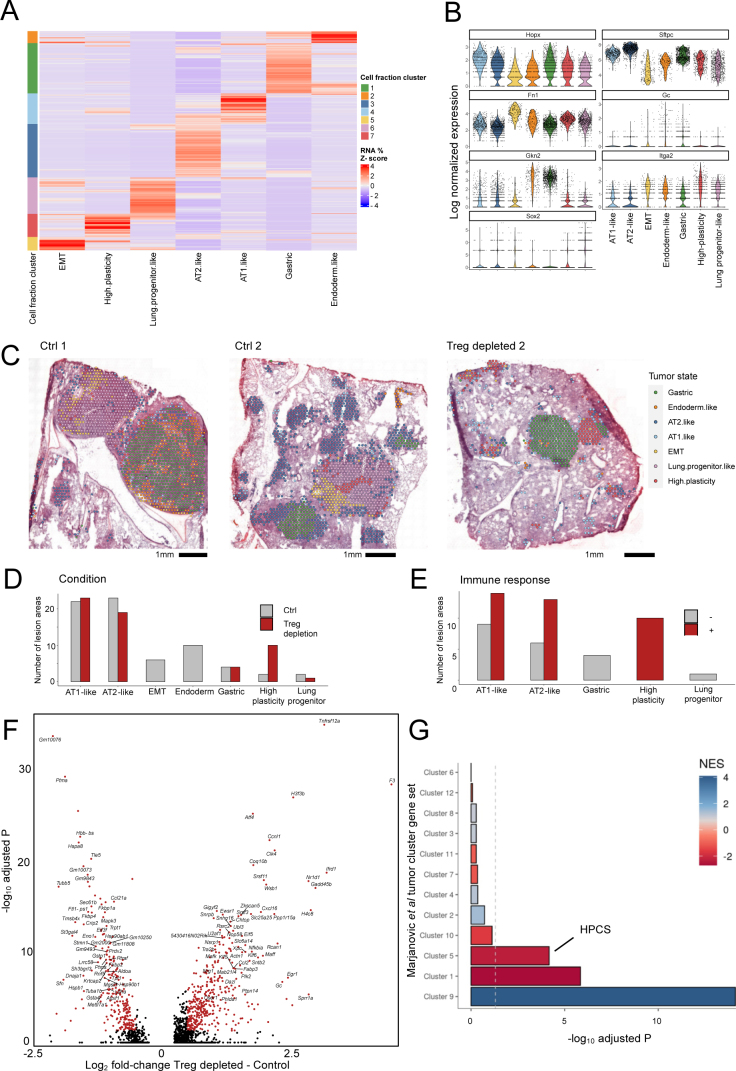
Spatial transcriptomic analysis of tumor cell states perturbed in response to Treg cell depletion. (**a**) Hierarchical clustering of tumor spots by tumor state RNA fractions. (**b**) Log normalized expression of tumor state marker genes in assigned spot clusters from A. (**c**) H&E staining of 3 independent KP tumor sections (in addition to those shown in Fig. 5b) with tumor spots denoted by their assigned cluster in A. (**d**) Number of tumor lesion areas identified across all lung tumor states in control or Treg depleted mice (85 tumor lesions total). (**e**) Number of tumor lesion areas identified in Treg depleted sections across all tumor states colored by immune response status in Treg depleted mice (*N* = 38 tumor lesion areas). (**f**) Differentially expressed genes (Wilcoxon test BH adjusted) between tumor cells between control and Treg depleted conditions. (*N* = 239 cells total). (**g**) GSEA of differentially expressed genes in F within gene sets defined by different tumor clusters identified in Marjanovic et al.^29^ which partially align with tumor states identified by deconvolution. Dashed line indicates adjusted *p*-values <0.05. (NES = normalized enrichment score; HPCS = high plasticity cell state). Analysis performed on (A-D, B-G) and images are representative of (C), one of two serial sections for each of four samples (DT and Ctrl two biological replicates each). One experiment was performed. Source data

In spots with tumors, the tumor-state fractions exhibited regional variation in gene expression programs, sometimes within seemingly the same tumor nodule (Fig. 5a). Tumor spots were clustered by their tumor-state fractions and typically showed a dominant tumor state in each spot (Extended Data Fig. 8a) manifested in the expression of corresponding marker genes (Extended Data Fig. 8b), forming continuous spatial patches of similar phenotypes (Fig. 5b and Extended Data Fig. 8c). Spots were grouped into tumor lesion areas, or contiguous patches of tumor cells belonging to the same cluster and quantified across control and T_reg_ cell-depleted conditions. Tumor states were also compared across tumors that had a detectable immune response (>10% of spots in IC or IFN signaling niches) or not in T_reg_ cell-depleted sections. T_reg_ cell depletion resulted in a pronounced increase in tumor lesion areas that corresponded to a high-plasticity cell state, specifically among tumor nodules associated with an immune response (Fig. 5c and Extended Data Fig. 8d,e)^29^. This was consistent with a significant enrichment of high-plasticity cell-state genes upregulated by tumor cells after T_reg_ cell depletion in scRNA-seq (Extended Data Fig. 8f,g and Supplementary Tables 13 and 14). Therefore, TME response to the removal of T_reg_ cells may elicit a gene program in LuAds that represents an unstable transitional state, which can give rise to other tumor states^28,29^. While IC and IFN niches were observed in the majority of tumor nodules after T_reg_ cell depletion, there were some nodules, and even areas within individual nodules, that did not (Fig. 4c). In particular, those with a gastric epithelial lineage gene expression program were selectively devoid of IC or IFN responses (Fig. 5c and Extended Data Fig. 8e). We assessed differential gene expression between immune response ‘rich’ and ‘poor’ lesion areas and found increased expression of *Gkn2* (gastrokine), *Pf4* (platelet factor 4/Cxcl4) and *Sox9* among other genes (Fig. 5d,e and Supplementary Table 15). Interestingly, *Sox9* expression in lung tumor cells was shown to enable their escape from NK cell-mediated killing in certain cases^27^, suggesting one potential mechanism of immune evasion. Similarly to a recent analysis of CRISPR-edited tumors^31^, the observed response to T_reg_ cell depletion was spatially restricted, as even ‘nonresponsive’ areas that were directly adjacent to responsive ones were deprived of immune cell or IC signals (Fig.6a,b). Therefore, regional variation in tumor state appears to define the TME response to T_reg_ cell depletion.

### Conserved T_reg_ cell-dependent features of human and mouse tumor microenvironment

**Fig. 7:**
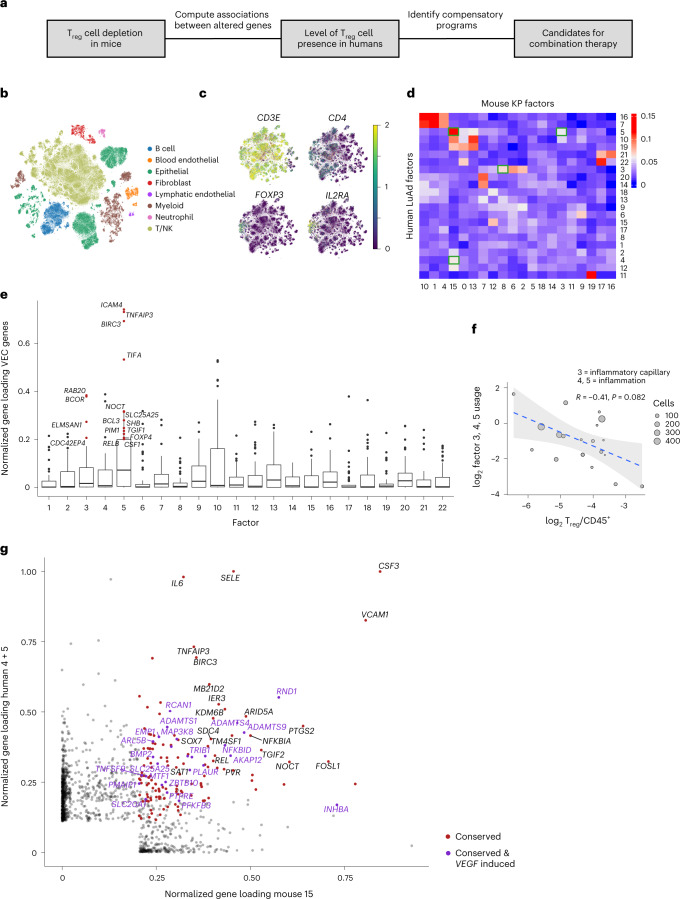
Factor analysis of T_reg_ cell ‘dependencies’ of accessory cell transcriptional states in human and mouse lung adenocarcinomas. **a**, Schematic of the experimental design. **b**, *t*-SNE plot of all cells (82,991 total cells) from 25 primary human LuAd or local metastases labeled by lineage. **c**, *t*-SNE of T/NK cell lineage colored by unique molecular identifier (UMI) counts of T_reg_ cell marker genes (maximum of two). **d**, Jaccard similarity between genes associated with mouse and human factors in tumor endothelial cells. Factors of interest with high correlation are highlighted by a green box. **e**, Conservation of activated VEC signature genes. Normalized gene loading (fraction of gene score across all factors) of genes within the mouse activated VEC signature across all human endothelial factors. Upper and lower notches of the box plot correspond to the 75th and 25th quartiles, respectively, and the middle notch corresponds to the median. Whiskers extend to the farthest data point no more than 1.5 times the interquartile range from the hinge, with outliers beyond that displayed as individual points. Select genes with high loadings of factors 3 and 5 are highlighted (*N* = 45 genes). **f**, Mean log_2_ sum of inflammation/angiogenesis associated human endothelial factor (3, 4 and 5) cell loadings plotted against log_2_ T_reg_ cell proportion in each human sample. Spearman correlation estimate (*R*) and *P* value are listed. Trend line represents a linear model fit between the two and shading indicating the 95% confidence interval (*N* = 19 human samples). **g**, Normalized gene scores (fraction of gene scores across all factors) in orthologous genes between mouse and human inflammation/hypoxia factors. Genes significantly attributed to both human factors and mouse factors are highlighted as conserved. VEGF-induced genes in endothelial cells were derived from the CytoSig database. Source data

**Extended Data Fig. 9:**
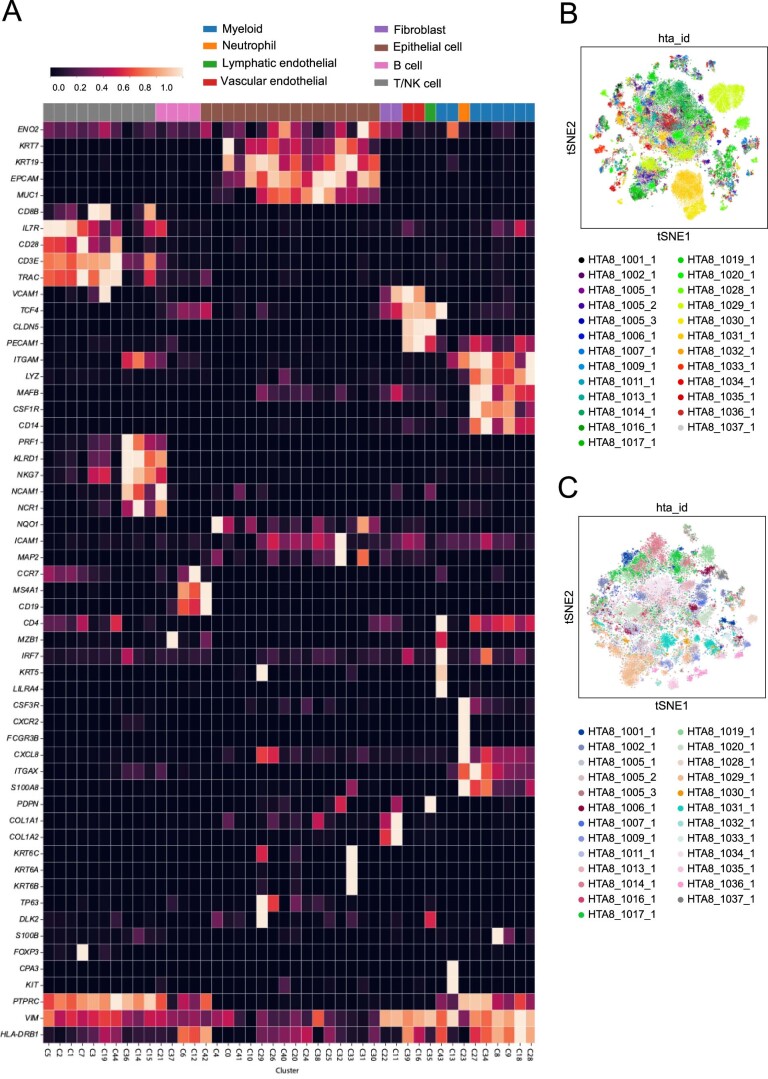
Clustering and cell linage annotation of scRNA-seq datasets of human lung adenocarcinomas. (**a**) Heatmap displaying genes used to determine lineage assignments for single cell PhenoGraph clusters in human LuAd samples. Color bar represents the mean log normalized gene expression in each PhenoGraph cluster scaled from 0 to 1 for each gene. (**b**) Global t-SNE embedding across of all human lineages as in Fig. 4b colored by sample. ID. All genes used for annotation are shown. (**c, d**) t-SNE embeddings of the T/NK lineage colored by PhenoGraph cluster (C) or sample ID (D). Source data

**Extended Data Fig. 10:**
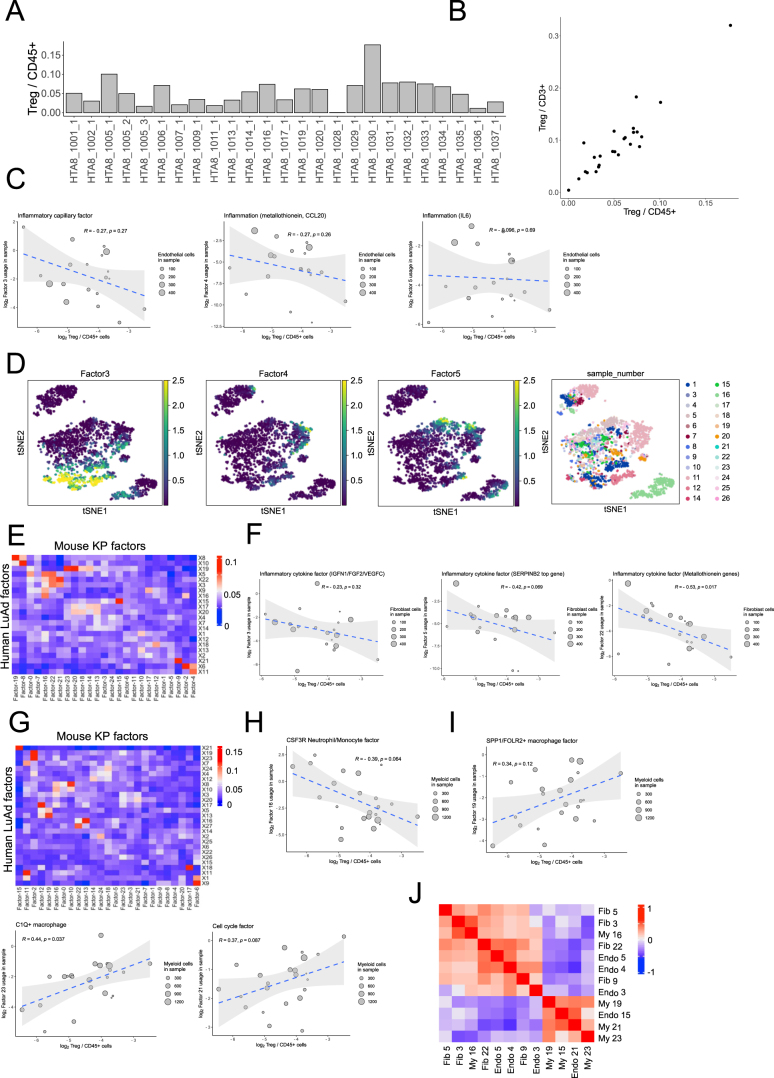
Association of Treg abundance with transcriptional features of endothelial cells in human LuAd and loadings of human and mouse fibroblast and endothelial cell factors. (**a**) Treg proportion of hematopoietic cells (CD45^+^) calculated from scRNA-seq data across all samples. (**b**) Treg proportion of hematopoietic cells compared to the Treg proportion of CD3^+^ cells across all human samples. (**c**) Mean log_2_ cell loading of CAR4^+^ capillary (factor 3) and other inflammation/hypoxia associated human endothelial factors (4,5) plotted against log_2_ Treg proportion in each patient sample. Spearman correlation estimate (R) and p value are listed. Trend line represents a linear model fit between the two and shading indicating the 95% confidence interval. (**d**) t-SNE of human endothelial cells colored by factor 3, 4, or 5 cell loading (max 2.5) or sample ID. (*N* = 19 patient samples). (**e,g**) Heatmap showing Jaccard similarity of genes associated with human and mouse fibroblast (E) or myeloid (G) factors. (**f,h,i**) Mean log_2_ cell loading of factors negatively associated with Treg frequency in fibroblasts (F) and myeloid cells (H), or positively associated in myeloid cells (I) plotted against log_2_ Treg proportion in each patient sample. Spearman correlation estimate (R) and p value are listed. Trend line represents a linear model fit between the two and shading indicating the 95% confidence interval. (fibroblast *N* = 20; myeloid *N* = 23). (**j**) Heatmap showing the Spearman’s correlation between Treg cell frequency associated human factors with conserved trends in mouse Treg-depletion. Source data

Next, we sought to test whether T_reg_ cell-dependent TME features observed in mice are conserved in human cancer (Fig. 7a) by leveraging variation in T_reg_ cell abundance in 25 primary or local metastatic LuAd samples from 23 individuals, analyzed using scRNA-seq (Supplementary Tables 16 and 17). Despite differences in composition and proportion of accessory cell types in these datasets, we were able to detect all cell populations corresponding to those observed in mice (Fig. 7b and Extended Data Fig. 9a,b). To determine whether the factors induced after T_reg_ cell depletion in mice are present in human LuAd samples with a low abundance of T_reg_ cells, we first determined the frequency of T_reg_ cells among all hematopoietic cells in each sample (Fig. 7c and Extended Data Figs. 9c and 10a,b). Next, we performed scHPF analysis for each of the cell lineages under investigation (Supplementary Table 18) and looked for orthologous genes shared between human and mouse factors to align gene programs between species (Fig. 7d). Then, we assessed the correlation of mean factor usage in single cells to T_reg_ cell frequency across human samples. This identified three factors negatively correlated with T_reg_ cell proportion that corresponded to aspects of the compensatory endothelial response to T_reg_ cell depletion in the KP mouse model (Extended Data Fig. 10c). The latter included factors whose most associated genes were related to activated aCap (*CAR4*, *CD36*, *IFNGR1*, *FAS*, *CX3CL1*, *TNFRSF11b*, *EDN1*; factors 3 and 5; Fig. 7e), inflammation and hypoxia (*VEGFB*, *PLAUR*, *SERPINE1*, *IL6*, *CXCL1*, *BCL3*, *PVR*, *IRF4*, *BATF3*, *TFP12*; factors 4 and 5) and angiogenesis factors (factor 3). We used the sum of these three factors as a general T_reg_ cell-responsive endothelial gene program to account for potential sample-specific, cell-type-specific or condition-specific effects that would separate a shared underlying biological program into separate factors during factorization (Extended Data Fig. 10d). Comparing this score to T_reg_ cell proportion, we observed a clear negative correlation—stronger than any factor individually—across tumor samples (Fig. 7f), which suggested conserved T_reg_ cell influence on this gene expression program. To further identify specific components of this shared T_reg_ cell-responsive endothelial gene expression program, we compared the loadings of genes in the factors related to inflammation and hypoxia across species (factors 4 + 5 in human LuAd, factor 15 in KP mouse; Fig. 7g). This identified genes encoding key inflammatory mediators (*IL6*, *CSF3*, *VCAM1*, *SELE*, *PTGS2*) and a host of VEGF-induced genes in endothelial cells (*RND1*, *ADAMTS1*, *ADAMTS4*, *ADAMTS9*, *AKAP12*) as conserved members of expression programs induced in endothelial cells in T_reg_ cell-poor TMEs across species.

Similar analyses of fibroblasts and myeloid cells also revealed corresponding T_reg_ cell-dependent mouse and human factors. For example, human fibroblast factors 3, 5 and 22 corresponded to IC mouse fibroblast factors 21 and 22, with overlapping genes including *IL6*, *IL1RL1*, *NFKB1*, *CCL2* and *LIF* (Extended Data Fig. 10e,f), while factor 9 (AP1 TF family members, *KLF2/4*, *SOX9*, *HES1*, *IRF1*) was negatively associated with T_reg_ cell proportion. Additionally, high usage of conserved CSF3R monocyte factor 16 (*CSF3R*, *PROK2*, *VCAN*) in human ‘T_reg_ cell-poor’ LuAd samples was consistent with the hypoxia, angiogenesis and NF-κB signaling related features (*VEGFA*, *HIF1A*, *CEACAM1*, *NOTCH1*, *BCL3*, *BCL6*) of this population in T_reg_ cell-depleted mice (Extended Data Fig. 10g,h and Extended Data Fig. 5d). Notably, several human myeloid factors and corresponding mouse factors showed positive correlation with T_reg_ cell presence, such as an *SPP1/FOLR2* macrophage factor, a cell cycle factor and a C1Q^+^ macrophage factor (C1Q, antigen presentation-related genes), which included genes encoding known negative regulators of innate and adaptive immunity (*CFH*, *CR1L*, *LAG3, PDCD1LG2*, *LILRB4*, *IL18BP*; Extended Data Fig. 10i). Interestingly, we observed similarly pronounced downregulation of this gene program upon T_reg_ cell depletion in both lung tumors and bleomycin-induced injury, suggesting that T_reg_ cells within both niches sustain certain immunomodulatory myeloid cell states. Further analysis of correlation between conserved T_reg_ cell-dependent human and mouse factors revealed a set of opposing TME programs (Extended Data Fig. 10j). One factor group in T_reg_ cell-poor or T_reg_ cell-deprived tumors included IL-1β/IL-18 signaling-related genes (*IL18RAP*, *IL1RAP*) expressed in angiogenic monocytes and tumor necrosis factor (TNF)/IL-1β-induced genes in fibroblast and endothelial cells involved in monocyte and neutrophil recruitment (*CSF3*, *CXCL1*, *CXCL2*, *CXCL8*, *CCL2*). The other, positively associated with T_reg_ cell presence, featured immunomodulatory genes that inhibit IL-1β/IL-18 signaling (*TMEM176B*, *IL18BP*; see Supplementary Table 19 for KP/injury/LuAd factors). These findings suggest a conserved role of T_reg_ cells in tuning transcriptional states of principal accessory cell types in the TME.

### Combinatorial T_reg_ cell depletion therapy

**Fig. 8:**
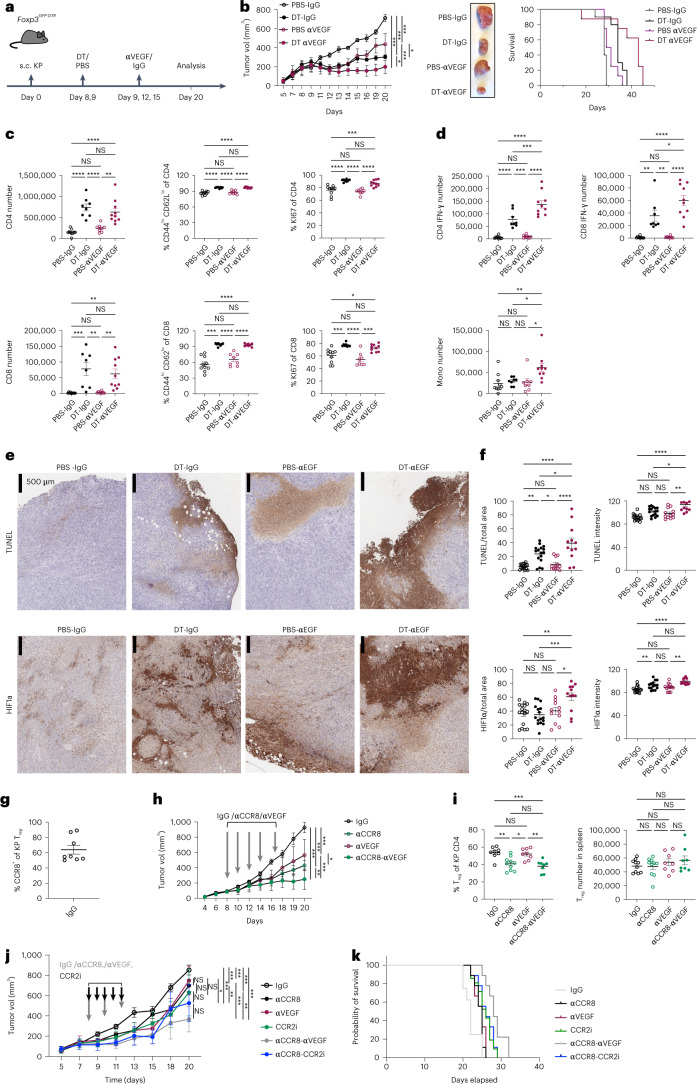
Systemic or intratumoral CCR8^+^ T_reg_ cell depletion combined with VEGF blockade restrains KP adenocarcinoma progression. **a**, Schematic of the experimental design; s.c., subcutaneous. **b**, Tumor growth dynamics upon the indicated therapeutic interventions. The data represent mean values of tumor volume measurements (left). Adjusted *P* values for day 20 measurements: PBS-IgG versus DT-IgG *P* < 0.0001; PBS-IgG versus PBS-αVEGF *P* = 0.0004; PBS-IgG versus DT-αVEGF *P* < 0.0001; DT-IgG versus PBS-αVEGF *P* = 0.0328; DT-IgG versus DT-αVEGF *P* = 0.0109; PBS-αVEGF versus DT-αVEGF; *P* = 0.0005. Representative image of tumor volumes at day 20 (center). Kaplan–Meyer survival curves followed by log rank (Mantel–Cox) of KP tumor-bearing mice (right). The ‘survival’ time reflects the end point of the experiment when tumor volume in individual mice reached 1 cm^3^; adjusted *P* values: PBS-IgG versus DT-IgG *P* = 0.0012; PBS-IgG versus PBS-αVEGF *P* > 0.05 (NS), PBS-IgG versus DT-αVEGF *P* = 0.0078; DT-IgG versus PBS-αVEGF *P* > 0.05 (NS); DT-IgG versus DT-αVEGF *P* = 0.05; PBS-αVEGF versus DT-αVEGF *P* = 0.0186. **c**,**d**, Quantification of the indicated immune cell subsets and frequencies of activated (CD44^hi^ CD62^lo^), proliferating (Ki67^+^) and IFN-γ-producing TCRβ^+^ CD4^+^ and TCRβ^+^ CD8^+^ cells in tumor samples shown in Fig. 8b in the indicated experimental groups of mice analyzed on day 20. **e**, Representative HIF1α and TUNEL staining of KP tumor sections. **f**, Quantification of HIF1α expression and apoptosis (TUNEL staining) in KP tumor sections; staining areas and signal intensity normalized by the total area and mean background intensity, respectively. 3–5 tumors from each experimental group were analyzed. (PBS-IgG *N* = 5; DT-IgG *N* = 4; PBS αVegf *N* = 3; DT αVegf *N* = 3) with four sections per individual tumor sample. Data represent the mean ± s.e.m. **g**, Proportion of intratumoral T_reg_ cells on day 20 after KP tumor transplantation. Data represent the mean ± s.e.m. of one of two independent experiments; *N* = 8. **h**, Tumor growth dynamics upon the indicated therapeutic interventions. Gray arrows indicate days of neutralizing antibody administration. The data represent mean values of tumor volume measurements (left). Adjusted *P* values for day 20 measurements: IgG versus αCCR8 *P* < 0.0001; IgG versus αVEGF *P* < 0.0001; IgG versus αCCR8-αVEGF *P* < 0.0001; αCCR8 versus αVEGF *P* = 0.0434; αCCR8 versus αCCR8-αVEGF *P* = 0.0044; αVEGF versus αCCR8-αVEGF *P* < 0.0001. **i**, Quantification of proportion and absolute numbers of intratumoral and splenic T_reg_ cells following treatment (left) and the corresponding T_reg_ cell numbers in spleens in the treated animals (right). Data in **h** and **i** represent the mean ± s.e.m. of one of two independent experiments, IgG *N* = 10, CCR8 *N* = 10, αVegf *N* = 8, CCR8-αVegf *N* = 8. **j**, Tumor growth dynamics upon the indicated therapeutic interventions (left). Gray and black arrows indicate timing of neutralizing antibody and CCR2 inhibitor (CCR2i) administration, respectively. The data represent the mean ± s.e.m. values of tumor volume measurements. Adjusted *P* values of day 20 measurements: IgG versus αCCR8 *P* = 0.0009; IgG versus αVEGF *P* < 0.0001; IgG versus CCR2i *P* < 0.0001; IgG versus αCCR8-αVEGF *P* < 0.0001; IgG versus αCCR8-CCR2i *P* < 0.0001; αCCR8 versus αVEGF *P* = 0.9982; αCCR8 versus CCR2i *P* = 0.6138; αCCR8 versus αCCR8-αVEGF *P* < 0.0001; αCCR8 versus αCCR8-CCR2i *P* = 0.0041; αVEGF versus CCR2i *P* = 0.9551; αVEGF versus αCCR8-αVEGF *P* = 0.0003; αVEGF versus αCCR8-CCR2i *P* = 0.0363; CCR2i versus αCCR8-αVEGF *P* = 0.0018; CCR2i versus αCCR8-CCR2i *P* = 0.2271; αCCR8-αVEGF versus αCCR8-CCR2i *P* = 0.4530. Plots include data from two independent experiments combined with nine animals in each group in experiment 1 (IgG *N* = 9, αCCR8 *N* = 9, αVEGF *N* = 9, CCR2i *N* = 9, αCCR8 *N* = αVEGF-9, αCCR8-CCR2i *N* = 9) and 4–6 animals per group in experiment 2 (IgG *N* = 4; CCR2i *N* = 6; CCR8-CCR2i *N* = 6). **k**, Kaplan–Meyer survival curves followed by Log-rank (Mantel–Cox) of KP tumor-bearing mice. The ‘survival’ time reflects the end point of the experiment when tumor volume in individual mice reached 1 cm^3^. Adjusted *P* values: IgG versus αCCR8 ***P < 0.0001; IgG versus αVEGF ***P < 0.0001; IgG versus CCR2i ***P < 0.0001; IgG versus αCCR8-αVEGF ***P < 0.0001; IgG versus αCCR8-CCR2i ***P < 0.0001; αCCR8 versus αVEGF *P* = 0.5687 (NS); αCCR8 versus CCR2i *P* = 0.7411 (NS); αCCR8 versus αCCR8-αVEGF ***P = 0.0002; αCCR8 versus αCCR8-CCR2i *P* = 0.0342; αVEGF versus CCR2i *P* = 0.8054 (NS); αVEGF versus αCCR8-αVEGF ***P = 0.0006; αVEGF versus αCCR8-CCR2i *P* = 0.0666 (NS); CCR2i versus αCCR8-αVEGF ***P = 0.0003; CCR2i versus αCCR8-CCR2i *P* = 0.6749 (NS); αCCR8-αVEGF versus αCCR8-CCR2i *P = 0.0489. Plots include data from two independent experiments combined with 5–11 animals in each group in experiment 1 (IgG *N* = 9, αCCR8 *N* = 9; αVEGF *N* = 9; CCR2i *N* = 11; αCCR8-αVEGF *N* = 9; αCCR8-CCR2i *N* = 5) and 4–10 animals per group in experiment 2 (IgG *N* = 7; CCR2i *N* = 10; CCR8-CCR2i *N* = 4). In **b**–**d**, **h** and **i**, plots are representative of one of two experiments with 8–10 mice per group each, at day 20 after transplantation. Number of mice per group in **b** and **c**: PBS-IgG *N* = 10; DT-IgG *N* = 10; PBS-αVEGF *N* = 9; DT-αVEGF *N* = 9; number of mice per group in **h** and **i**: IgG *N* = 10; αCCR8 *N* = 10; αVEGF *N* = 8; αCCR8-αVEGF *N* = 8. Source data

These results highlighted candidate compensatory pathways, whose targeting in combination with current clinical-stage intratumoral T_reg_ cell depletion strategies^32,33^ could improve therapeutic efficacy. In this regard, the increased expression of VEGF pathway-related genes upon T_reg_ cell deprivation was of particular interest suggesting that heightened VEGF signaling may ‘buffer’ the negative impact of T_reg_ cell depletion on the tumor-supporting TME function and facilitate a rebound in the tumor progression. We tested the above possibility by investigating whether combining short-term T_reg_ cell depletion with VEGF blockade can lead to an improved control of KP tumor progression. We transplanted KP adenocarcinomas into *Foxp3*^GFP-DTR^ mice and administered them with DT and mouse VEGF-A neutralizing antibody (aVEGF) after tumors became macroscopically detectable (Fig. 8a). While T_reg_ cell depletion and VEGF blockade alone could slow tumor progression, their combination had a markedly more pronounced therapeutic effect (Fig. 8b). Assessment of the overall survival rate, when mice were left untreated after the initial response and killed after rebound (tumor volume reached 1 cm^3^, maximum allowed by the institutional guidelines) showed that combination therapy improved survival in comparison to either monotherapy or untreated groups (Fig. 8b). While similarly increased numbers and activation level of tumoral T cells were observed in ‘DT + aVEGF’ and ‘DT-only’ in comparison to ‘aVEGF-only’ tumor samples on day 20 of transplantation (Fig. 8c), IFN-γ-producing CD4^+^ T cells and IFN-γ-producing and TNFα-producing CD8^+^ T cells were markedly increased in the combination treatment group as were monocyte numbers (Fig. 8d). Moreover, we observed further increases in tumor hypoxia and apoptosis upon combined T_reg_ cell depletion and VEGF blockade in comparison to both monotherapeutic modalities and untreated control groups (Fig. 8e,f). Notably, KP tumors failed to respond to PD-1 blockade, which did not offer additional therapeutic benefits when combined with VEGF blockade in full agreement with a recent study of antiangiogenic, anti-PD-1 and chemotherapy in a KP lung cancer model^34^. Recent studies revealed high amounts of chemokine receptor CCR8 displayed by T_reg_ cells in human cancers^35,36^ highlighting their depletion as a therapeutic strategy^33,37,38^. Thus, we examined the therapeutic potential of short-term VEGF blockade combined with antibody-mediated depletion of CCR8-expressing T_reg_ cells, which represented only a fraction of intratumoral T_reg_ cells in KP tumors (Fig. 8g). While CCR8 antibody treatment alone diminished tumor growth, a markedly more pronounced effect was observed when it was combined with VEGF blockade (Fig. 8h). Notably, this regimen was associated with a mere 15% decrease in overall population of tumor-associated T_reg_ cells in the absence of their noticeable changes in the secondary lymphoid organs (Fig. 8i). Besides VEGF-A, whose neutralization was conducted as a proof-of-concept approach for the discovery of orthogonal combination therapy, we noted additional candidate compensatory pathways enriched in the T_reg_ cell-poor or cell-depleted TME including the CCR2–CCL2 axis, inhibitors of which are currently tested as monotherapies or combination therapies of human cancers. To further test the utility of assessment of early TME responses to T_reg_ cell depletion for identifying combinatory therapeutic modalities, we subjected KP tumor transplanted mice to a similar short-term treatment with CCR8 antibody and a selective CCR2 antagonist RS-504393 (CCR2i). The latter combination provided minimal additional therapeutic benefit in comparison to anti-CCR8 and CCR2i monotherapies contrary to aVEGF/CCR8 combination (Fig. 8j,k). These results suggest that CCR2 blockade and T_reg_ cell depletion may converge on shared or partially overlapping TME states, whereas VEGF blockade offers an orthogonal intervention and highlights potential for discovery of orthogonal cancer therapies through single-cell and spatial analyses of early TME responses to acute perturbation.

## Discussion

Successes in therapeutic targeting of PD-1 and CTLA-4 pathways in T lymphocytes are viewed as clinical evidence supporting the notion of cancer surveillance by cells of the adaptive immune system akin to that of pathogens. On the other hand, a growing realization of the important roles immune cells play in supporting normal tissue function, maintenance and repair suggests an alternative, even if not mutually exclusive view of tumor–immune interactions. Within the latter framework, the TME can be considered as a tissue-supporting multicellular network, which in response to cues emanating from cancerous cells supports their growth. In this regard, cancer represents a special state of a parenchymal cell, whose support by both immune and non-immune cells is guided by common, yet poorly understood principles of tissue organization. T_reg_ cells suppress immune responses directed against self-antigens and foreign antigens to protect tissues from inflammation-associated loss of function^1^. Besides this indirect tissue-supporting functionality, T_reg_ cells were also implicated in direct responses to injury and other forms of tissue damage through production of tissue repair factors^16,39–42^ suggesting that these functions of T_reg_ cells are conserved. Furthermore, T_reg_ cells were shown to support skin and hematopoietic stem cell niches^15,43,44^. Therefore, it is reasonable to assume that in human solid organ malignancies and in experimental mouse cancers T_reg_ cells likely play similar dual roles supporting tumor growth-promoting accessory cell states.

Here, we showed that T_reg_ cells have a profound impact on states of key accessory cells in a genetic autochthonous mouse model of NSCLC, in an experimental model of lung injury and in human LuAds. Using robust unsupervised data-driven computational analyses, we found that T_reg_ cells support conserved gene programs—factors—across experimental models of lung cancer and injury, suggesting their role in coordinating broad, shared accessory cell functions that extend to various conditions and tissue states. The latter included human immunomodulatory C1Q^+^ (*CFH*, *CR1L*, *LAG3*, *PDCD1LG2*, *LILRB4*, *IL18BP*) and *SPP1/FOLR2* macrophage factors and their mouse counterparts, which were positively associated with the T_reg_ cell presence. A similar macrophage gene program is also reported to be enriched in NSCLC lesions^45^ and sustained by T_reg_ cells in mouse models of melanoma and breast cancer^46^.

Our analysis of the distribution of activated cell types and gene expression programs with respect to their localization within and around tumor nodules showed high concordance of characteristic gene programs that were identified by scRNA-seq and ST analyses in situ. Notably, T_reg_ cell depletion induced the IC response program localized to tumor nodule cores, while the IFN response program was most notable in the margins of tumor foci. The display of these programs by multiple cell types present within the same local niche suggests that they are elicited by common signals (‘signal niche’), for example, hypoxia response in the tumor nodule cores and a transient burst of IFN-γ produced by CD8^+^ T cells and NK cells concentrated in the tumor margins^47^. We also observed heterogeneity between T_reg_ cell depletion responsive and nonresponsive tumor foci distinguished by the presence or paucity of the IC gene program. Interestingly, tumor nodules that failed to induce the IC gene program in response to T_reg_ cell depletion expressed Sox9 in agreement with a recent study where upregulation of Sox9 in human LuAd conferred resistance to NK cells^27^.

Among the conserved gene programs negatively associated with T_reg_ cell presence in mouse and human lung cancer, we noted the VEGF signaling pathway. This included increased expression of VEGF signaling-related genes in endothelial cells and increased expression of VEGF-A in myeloid and other cell types. This most likely reinforces the immunosuppressive TME providing support for tumor growth consistent with a recent report of tumor ischemia caused by the transient spike in intratumoral IFN-γ following CD25 antibody photoimmunotherapy-induced T_reg_ cell depletion^47^. In addition, lung EC-derived VEGF was shown to specify development of CAR4^hi^ endothelial cells and promote vascularization and tissue regeneration following injury^48,49^. VEGF has also been suggested to exert an immunomodulatory effect on cells of the innate and adaptive immune system^50^. Considering VEGF targeting being an approved therapy for some human cancers, combined VEGF-A and T_reg_ cell targeting serves as a proof-of-concept for a rational combination therapy instructed by the new knowledge of TME transcriptional connectivity. While near complete loss of the T_reg_ cell pool in KP-DTR mice led to systemic autoimmunity and inflammation, VEGF blockade coupled with CCR8 antibody-mediated depletion of intratumoral T_reg_ cells showed impressive therapeutic efficacy with no adverse effects. The latter owes to the fact that CCR8 expression is selectively enriched in highly activated intratumoral T_reg_ cell subsets in human and mouse malignancies^35,36,38^. Our observation that a combination of CCR8 antibody-mediated intratumoral T_reg_ cell depletion with CCR2 blockade did not yield additional benefit in comparison to the corresponding monotherapies suggests that the latter either directly or indirectly converge on a shared regulatory node and highlights the utility of preclinical selection of combinatorial therapeutic strategies informed by scRNA-seq and ST analyses of early TME responses.

Our study highlights a generalizable approach where perturbation of a given cell population in an engineered genetic cancer model enabled computational learning of its ‘connectivity’ and influence on the TME and other diseased tissue states, which could then be compared to the human clinical settings. A surfeit of secreted and cell surface molecules has been implicated in T_reg_ cell-mediated immunosuppressive and tissue-supporting functions. However, none of these individual modalities can predominantly account for the bulk of these functionalities. Combinatorial targeting of these putative mediators will enable elucidation of the molecular mechanisms of the observed T_reg_ cell dependencies in the TME. Our results suggest that T_reg_ cells serve as an essential component of a complex network of accessory cells of both hematopoietic and non-hematopoietic origin. Shared perturbations in their transcriptional states observed across the three different settings imply that the identified interdependencies of T_reg_ cells and other components of tissue-supporting cellular networks are conserved and can be exploited to develop new strategies for rational therapies of cancer and other diseases.

## Methods

### Experimental model and mouse details

#### Mice

Animals were housed at the Memorial Sloan Kettering Cancer Center (MSKCC) animal facility under specific pathogen-free conditions according to institutional guidelines. All studies were performed under protocol 08-10-023 and approved by the MSKCC Institutional Animal Care and Use Committee. Mice used in this study had no previous history of experimentation or exposure to drugs. *Foxp3*^GFP-DTR^ and *Kras*^LSL-G12D^ *Trp53*^fl/fl^ mice were previously described^10,13^. Adult male and female mice (6 weeks or older) were used for all experiments.

#### Lung adenocarcinoma and bleomycin-induced fibrotic injury induction

Cre recombinase-mediated induction of KP LuAds was previously described^10^. Briefly, mice were anesthetized with a 160–180 μl ketamine–xylazine mixture and infected with Cre recombinase-expressing adenovirus (1 × 10^8^ plaque-forming units) via intratracheal administration. Tumors developed within approximately 3 months. For the induction of fibrotic injury, pharmaceutical-grade bleomycin (Fresenius Kabi) was administered intranasally to anesthetized mice (0.06 U per mouse). For the s.c. KP tumor growth model, KP cells were resuspended in sterile PBS and injected to the flank subcutaneous space (1 × 10^6^ KP cells in 200 μl per mouse).

#### Diphtheria toxin, VEGF, PD-1, CCR8 antibody and CCR2i treatments

DT (List Biological Laboratories) was administered to mice (1 μg per mouse in PBS) via retro-orbital injection twice on two consecutive days. For tumor transplantation experiments, DT was injected on days 8 and 9 after tumor s.c. transplantation. Mouse polyclonal neutralizing VEGF-A antibody (R&D clone AF-493-M) or control IgG (BioXcell clone BE0130) were injected on days 9, 12 and 15 (20 μg per mouse per injection) with or without DT, or on days 8, 10, 12, 14 and 17 with or without CCR8 antibody (BioLegend clone SA214G2; 240 μg per mouse per injection). PD-1 antibody (BioXcell clone BE0146) alone or in combination with VEGF-A antibody was administered on days 8, 10, 12, 14 and 17 (250 μg per mouse per injection). RS-504393 CCR2 inhibitor (CCR2i; 2517, Tocris) was administered (50 mg per kg body weight) daily in combination with CCR8 antibody, VEGF-A antibody or their combination. In these experiments, CCR8 and VEGF-A antibodies were administered on days 8, 10 and 12, and CCR2i was administered daily starting on day 8 and ending on day 12.

#### Human lung adenocarcinoma samples

Individuals with LuAd undergoing a surgical resection or tissue biopsy at MSKCC were identified and biospecimens collected prospectively from 2017 to 2020. All participants from whom biospecimens were obtained provided informed consent for an MSKCC-wide biospecimen collection and analysis protocol. Recruitment was designed to capture a wide, unbiased swath of heterogeneous disease, with a slight emphasis on EGFR-mutated tumors with a high propensity to transform to more aggressive subtypes. Biases may be present related to this recruitment design, the race, sex, smoking status and the general population of MSKCC. Use of all participant material and data described in this paper was performed under ethical approval obtained from the MSKCC Institutional Review Board (study nos. 06-107 and 12-245). Only continuous trends between cell proportion and factor use were assessed across all participants and therefore controls based on sample groupings are not relevant.

#### Cell isolation and flow cytometry

For isolation of immune and stromal cells, lungs were perfused, placed into 5 ml microcentrifuge tubes containing 400 μl of cold serum-free RPMI and chopped with scissors (1–2 mm). Lung fragments were placed in 2–3 ml of pre-warmed digestion medium (RPMI 1640, 10 mM HEPES buffer pH 7.2 to 7.6, 1% penicillin–streptomycin, 1% l-glutamine, liberase (Sigma-Aldrich, 05401020001) and 1 U ml^−1^ DNase I (Sigma-Aldrich, 10104159001; 2–3 ml)) and incubated for 30 min at 37 °C. After digestion, supernatant was collected and cells were resuspended in ice-cold RPMI 1640 containing 5% FCS (Thermo Fisher, 35010CV), 1 mM HEPES pH 7.2 to 7.6 (Corning, MT25060CI), 1% penicillin–streptomycin (Corning, MT30002CI) and 200 mM l-glutamine (Corning, MT25005CI). After additional digestion for 1 h of the remaining tissue, both digested cell fractions passed through a 100-μm strainer (Corning, 07-201-432), washed and FACS sorted. For cell isolation from transplanted KP tumor-bearing mice, tumors were placed into 5 ml microcentrifuge tubes containing 400 μl of cold serum-free RPMI 1640, chopped with scissors and incubated in digestion medium containing 1 mg ml^−1^ collagenase (Sigma, 11088793001) and 1 U ml^−1^ DNase I (Sigma-Aldrich, 10104159001) and beads on a shaker at 37 °C for 1 h. For cytokine production measurements, cells were incubated at 37 °C, 5% CO_2_ for 3 h in the presence of 50 ng ml^−1^ phorbol-12-myristate-13-acetate (Sigma-Aldrich, P8139), 500 ng ml^−1^ ionomycin (Sigma-Aldrich, I0634), 1 μg ml^−1^ brefeldin A (Sigma-Aldrich, B6542) and 2 μM monensin (Sigma-Aldrich, M5273). Cells were stained with Ghost Dye Red 780 (Tonbo Bioscience, 13-0865) or Zombie NIR Flexible Viability Kit (BioLegend, 423106) and a mixture of fluorophore-conjugated antibodies for 30 min at 4 °C cells, washed and fixed with 1% paraformaldehyde (Electron Microscopy Sciences, 15710). For intracellular staining, cells were fixed and permeabilized with the BD Cytofix/Cytoperm Kit or with the Thermo Fisher Transcription Factor Fix/Perm Kit according to the manufacturer’s instructions and analyzed on a BD LSR II flow cytometer or sorted on a BD Aria II flow cytometer. Post-sort cell purity was routinely higher than 95%. Flow cytometry data were collected on an LSR II using FACS Diva v8.0 (BD), or on Aurora using SpectroFlo v2.2.0.3 (Cytek). Flow cytometry data were analyzed using FlowJo v 10.6.1 (BD).

#### Immunofluorescence microscopy, histological and spatial transcriptomic analyses

Perfused lungs were fixed for 1 h at 22 °C in 4% paraformaldehyde and dehydrated at 4 °C in 30% sucrose, snap-frozen in OCT compound (Sakura Tissue-Tek, 4583). For ST, samples were flash frozen without fixation. All samples were sectioned with a Leica CM1950 Cryostat at −2 °C, to a thickness of 10 μm. Sections were fixed in acetone for 20 min at −20 °C, rehydrated in PBS, blocked with 10% normal donkey serum (Jackson ImmunoResearch, 017-000-121) in PBS, 0.3% Triton X-100, and stained overnight with fluorophore-conjugated antibodies at 4 °C in a humidified chamber. Thereafter, nuclei were stained with DAPI (5 mg ml^−1^; Abcam, 28718-90-3) or Draq7 (5 μM; Abcam, 109202) for 20 min at 22 °C. Sections were imaged in SlowFade mounting medium (Life Technologies, S36938) using a confocal Leica SP8 microscope. For histology, tissues were fixed in 10% neutral buffered formalin, embedded in paraffin, and sectioned. For the TUNEL assay, sections were processed under standardized conditions using the DeadEnd Fluorometric Detection System (Promega, G3250), and subsequent immunohistochemistry was carried out using BOND Polymer Refine Detection Kit (Leica, DS9800), according to the manufacturer’s instructions. All Images were processed and analyzed using ImageJ package v2.0.0-rc-69/1.52p. Distances between cells of interest were quantified following the same strategy and using similar code as described elsewhere^51^.

#### Antibodies

See Supplementary Table 20 for all antibodies used in this study.

#### RNA-seq library preparation and sequencing

Cell populations were sorted straight into TRIzol (Thermo Fisher, 15596018), RNA was precipitated with isopropanol and linear acrylamide, washed with 75% ethanol, and resuspended in RNase-free water. After RiboGreen quantification and quality control by Agilent BioAnalyzer, 0.4–2.0 ng total RNA with RNA integrity numbers ranging from 1.0 to 9.9 underwent amplification using the SMART-Seq v4 Ultra Low Input RNA Kit (Clonetech, 63488), with 12 cycles of amplification. Subsequently, 1.5–10 ng of amplified cDNA was used to prepare libraries with the KAPA Hyper Prep Kit (Kapa Biosystems, KK8504) using 8 cycles of PCR. Samples were barcoded and run on a HiSeq 4000 or HiSeq 2500 in rapid mode in a 50 bp/50 bp paired-end run, using the HiSeq 3000/4000 SBS Kit or HiSeq Rapid SBS Kit v2 (Illumina). An average of 32 million paired reads were generated per sample, and the percentage of mRNA bases per sample ranged from 62% to 88%.

#### RNA-seq analysis

Paired-end RNA-seq reads were mapped to the genome using STAR^52^ v2.7.3a. Gene annotations were downloaded from Ensembl release 83, which is based on mouse genome assembly GRCm38. R v3.6.0 was used for generating count matrices and DESeq2 (ref. ^53^) was used for principal-component analysis, to identify DEGs and for Spearman correlations calculations and for hierarchical clustering and generation of *k*-means heat maps.

#### Single-cell RNA sequencing

Single-cell RNA-seq was performed on FACS-sorted mouse lung KP cells or human LuAd samples, on the Chromium instrument (10X Genomics) following the user guide manual (CG00052 Rev E) for 3′ v2 and v3 as previously described^54^. Briefly, sorted cells were washed once with PBS containing 0.04% BSA and resuspended in PBS containing 0.04% BSA to a final concentration of 700–1,200 cells per μl. Viability of cells was confirmed to be above 80%, as confirmed with 0.2% (wt/vol) Trypan Blue staining (Countess II). Then samples were encapsulated in microfluidic droplets at a dilution of ∼70 cells per ml (doublet rate ∼3.9%). Encapsulated cells were subjected to a reverse transcription (RT) reaction at 53 °C for 60 min. After RT, the emulsion droplets were broken and barcoded cDNA was purified with DynaBeads MyOne SILANE, followed by 14 cycles of PCR amplification (98 °C for 180 s; (98 °C for 15 s, 67 °C for 20 s, 72 °C for 60 s) × 12 cycles; 72 °C for 60 s). Then, 50 ng of PCR-amplified barcoded cDNA was fragmented with the reagents provided in the kit and purified with SPRI beads to obtain an average fragment size of 600 bp. Next, the DNA library was ligated to the sequencing adaptor followed by indexing PCR (98 °C for 45 s; (98 °C for 20 s, 54 °C for 30 s, 72 °C for 20 s) × 10 cycles; 72 °C for 60 s). An average of 5,000 cells were targeted for each tumor sample. The resulting DNA library was double-size purified (0.6–0.8×) with SPRI beads and sequenced on an Illumina NovaSeq platform (R1: 26 cycles (KP), 28 cycles (LuAd); i7: 8 cycles; R2: 96 cycles (KP), 90 cycles (LuAd)) resulting in 184.5–186.1 million reads per sample (average reads per single cell, 42,000; average reads per transcript, 4.40–7.14; KP).

Visium spatial gene expression slides were permeabilized at 37 °C for 12–18 min and polyadenylated. mRNA was captured by primers bound to the slides. RT, second-strand synthesis, cDNA amplification and library preparation proceeded using the Visium Spatial Gene Expression Slide & Reagent Kit (10X Genomics PN 1000184) according to the manufacturer’s protocol. After evaluation by real-time PCR, cDNA amplification included 11–12 cycles; sequencing libraries were prepared with 8 cycles of PCR. Indexed libraries were pooled equimolar and sequenced on a NovaSeq 6000 in a PE28/120 run using the NovaSeq 6000 S1 Reagent Kit (200 cycles; Illumina). An average of 219 million paired reads were generated per sample.

#### Computational analysis of scRNA-seq data

For basic pre-processing and lineage identification see Supplementary Methods. We performed dimensionality reduction using principal-component analysis (specifying 50 principal components (PCs); nPC = 50), then visualized the data in two dimensions 2D using *t*-SNE on the PCs (perplexity parameter set to 50 (KP) or 100 (injury)). The cells were grouped into clusters using PhenoGraph^18^ on the PC space, with *k* = 30 (Extended Data Fig. 2b). We established that clustering was robust to slight changes in *k*, by reclustering the cells under varying *k* (*k* ${\it{\epsilon }}$ (20, 25, 30, 35, 40, 45)) and measuring consistency using the adjusted Rand index (using the sklearn package in Python), obtaining an average Rand index > 0.85. To annotate each cluster as a specific lineage, we computed the average expression of known lineage markers (Extended Data Fig. 2c,d). All the genes used for annotation are listed in the heat map^48,55–60^.

For human LuAd samples, non-empty droplets were defined using CellBender on a per-sample basis^61^. The expected number of cells was defined by SEQC output after the initial quality filters described above, plus 25,000, to ensure an adequate number of empty droplets in each human sample. A learning rate of 0.0001 (modified to 0.00005 for samples needing a slower learning rate) was used with 300 epochs. Viable cells were identified with a library size greater than 500 UMIs, gene number greater than 250, log_10_ genes per UMI greater than 0.8 (complexity), and less than 20% mitochondrial transcripts.

UMI counts from non-empty droplets with doublets removed were normalized by first dividing by the library size (UMI counts per droplet), multiplying by a scale factor of 10,000, and then taking the natural logarithm of 1 + the normalized counts. Before dimensionality reduction and clustering, genes were filtered out if they were detected in less than 10 cells, had low transcript annotation quality (transcript support level 4 or 5 in Ensembl 85), or belonged to categories including mitochondrial transcripts, highly expressed ncRNAs, ribosomal RNAs, immunoglobulin transcripts, hemoglobin genes or T cell antigen receptor variable regions. This resulted in 18,597 retained genes and 84,909 cells. The median total counts and number of cells per sample are listed in Supplementary Table 11.

##### Doublet detection

For all mouse model samples, we performed doublet detection using Scrublet^62^ with default parameters (that is, expected_doublet_rate = 0.06, min_counts = 2, min_cells = 3, min_gene_variability_pctl = 85, log_transform = true, n_prin_comps = 30) on each sample individually. Since we were more interested in analyzing specific lineages, we removed doublets when processing each lineage individually (as described below).

In human samples, doublets were identified on non-empty droplets for each sample individually using DoubleDetection (10.5281/zenodo.2678041) with a *P*-value threshold of 1 × 10^−7^ and a voter threshold of 0.8. This algorithm was used because of its higher relative accuracy among doublet detection methods^63^, important for consistency across heterogeneous sample mixtures. Doublets were removed before lineage identification.

##### Density plots

For analysis of individual lineages (mouse samples), see Supplementary Methods. *t*-SNE plots are valuable to build a hypothesis but it can be difficult to glean the density of cells from different conditions due to cells (dots) overlapping on top of each other. To complement the *t*-SNE plots (colored by conditions such as Fig. 2b,e) and further highlight the finding that T_reg_ cell depletion has different effects in different cell populations, we chose to represent the distribution of the cells in the *t*-SNE plot using a density plot. We used the kdeplot implementation in Seaborn package in Python (with non-default parameter thres = *0*).

##### scRNA-seq differential expression

Differential expression testing between tumor cells of control or DT conditions was performed using MAST^64^ on log-normalized values. Only genes in at least 10% of cells in either condition and a minimum log fold change of 0.25 (2,654 genes) were used as input. Significant genes were defined as adjusted *P* value < 0.05 and log fold change > 0.5. Gene-set enrichment analysis was performed using fgsea^65^ with log fold-change values of significant genes. Gene sets derived from tumor clustering in work by Marjanovic et al.^29^ were used to assess enrichment (Extended Data Fig. 8g; scrna_tumor_de_fgsea).

##### Milo analysis

Milo incorporates information from biological replicates to assign a *P* value for fold changes in neighborhood cellular abundance between experimental conditions, where neighborhoods are defined as regions of transcriptionally similar cells in a *k*-nearest neighbor (kNN) graph generated. This method provided us with rigorous statistics to compare the frequency of different transcriptional states between conditions.

For each lineage, we sought to quantify the changes in density of control and DT cells in each neighborhood in the kNN graph using Milo^19^. Conceptually, Milo is analogous to differential gene expression analysis, but instead of identifying genes that are differential between two groups of cells, Milo tests for differential cell density in (possibly overlapping) neighborhoods in the kNN graph, across different conditions. Milo also considers the originating sample of each cell and treats any batch effect as a covariate. However, since we did not observe significant batch effects in our data, the design matrix we supplied only included the sample identity and experimental condition of each cell.

To perform the analysis, we first constructed a kNN graph (*k* = 30) on PCs using the buildGraph function in Milo. For each lineage, we used the same number of PCs (nPCs = 50) as for clustering and cell-type annotation above. We constructed neighborhoods on top of the kNN graph using the Milo makeNhoods function with default parameters (prop = 0.1, refined = true), then counted cells in each neighborhood using the countCells function and assessed statistical significance using testNhoods and calcNhoodDistance for spatial FDR correction. We used default parameters in all these cases. Results were then visualized using the plotNhoodGraphDA function with alpha set to 1 in all cases (implying that neighborhoods with spatial FDR < 1 are colored in all visualizations). We further assigned each neighborhood to a cell type if more than 80% of the cells in it belonged to that cell type; otherwise, the neighborhood was termed ‘mixed’.

##### Factor analysis

To identify gene programs and their usage across cells, we used the scHPF package^21^. scHPF is a Bayesian factorization method that explicitly models sparsity in scRNA-seq count data, using hierarchical Poisson factorization to achieve positive-valued loadings across a selected number of factors for individual cells and genes. The method provides gene scores, which assign each gene a score for gene membership in a factor, and cell scores, which quantify the usage of each factor by a cell. Cells with high cell scores for a factor will use the gene program represented by that factor at higher levels; the gene program, in turn, consists of genes with high gene scores for that factor. In the context of response to T_reg_ cell perturbation in cells from different lineages, scHPF provided an ideal unsupervised and data-driven way to extract gene programs (factors) that are systematically altered by the perturbation.

In the mouse tumor samples, scHPF was run using default hyperparameters in the endothelial, fibroblast and myeloid lineages to obtain 20 endothelial-specific factors, 25 fibroblast-specific factors and 25 myeloid-specific factors.

##### Differential factor usage between diphtheria toxin and control

We expect that the coordinated gene program response to the impact of T_reg_ cell depletion should reflect as factor cell scores being differential between control and DT conditions. To quantify this, we computed the average cell score of every factor in each cluster of cells (grouped by the cell type they belong to) for each condition. This result is presented as a heat map in Fig. 2i for endothelial cells, Extended Data Fig. 5a for fibroblasts, Extended Data Fig. 5c for myeloid cells in the tumor model and Fig. 3g for endothelial cells in the bleomycin injury model. Investigating averages at the cluster level ensures that any factors that reflect subtle shifts in cell states within a cell type will be identified. We then studied those factors that have higher averages in DT compared to control.

To ensure that our factors are significantly differential between control and DT, we considered cell scores for each factor in each cluster and computed *P* values between the two conditions using a Mann–Whitney *U* test as implemented in the scipy.stats.mannwhitneyu package in Python. The *P* values are reported in Supplementary Table 6. We then considered factors that were robust to random initialization of scHPF (Supplementary Fig. 1 and ‘Robustness analysis of factors’), were biologically relevant and had *P* values < 0.01 for further analysis.

##### Robustness analysis of factors

We assessed the robustness of the obtained factors in two ways. First, we sought to ensure that the obtained factors were robust to random initializations. For this, we fixed the number of factors computed and reran the model for 20 iterations. To quantify the similarity across iterations, we computed Pearson correlation (for both gene and cell scores) between best matching factors between iterations. The best matching factors between any two iterations were identified using an implementation of the Hungarian^66^ matching algorithm. The algorithm matches each factor from one iteration to the best matching factor from a second iteration such that the total cost is minimized, where the cost is defined as (1 − pairwise correlation score between two iterations). We used the Python (v3.8) implementation of the linear_sum_assignment function in the optimize module of SciPy package (v1.7.1). After matching, we reported the median correlation score between pairs of iterations (Supplementary Fig. 1).

Second, we sought to ensure that the factors we identified in our analysis (highlighted in red in Figs. 2i and 3g and Extended Data Fig. 5a, c) as being different between control and DT conditions (‘Differential factor usage between diphtheria toxin and control’) were robust to changes in parameters, mainly the choice of number of factors. This test ensures that the obtained factors were not identified by chance and that they constitute robust signal in the data. For this, we fixed the number of factors computed above (that is, 20 factors for endothelial, 25 factors for fibroblasts and 25 factors for myeloid) as the baseline. Then, we reran scHPF for a range of number of factors (around the chosen value) and computed correlations with the specific factors of interest. To compute the correlation to the best matching factor, we used the same strategy of the Hungarian matching algorithm as described above. The average correlation over 20 such iterations was then reported (Supplementary Fig. 1).

We repeated the same computation to assess the robustness of chosen factors in the bleomycin injury model.

##### Comparison of human and mouse factors

In human samples, scHPF was run with default hyperparameters and ten random initializations in the endothelial, fibroblast and myeloid lineages, using raw UMI counts for genes expressed in at least 1% of cells within the lineage. This left 12,533 genes in the endothelial lineage, 13,216 genes in the fibroblast lineage and 12,253 genes in the myeloid lineage for factor analysis. To select the number of factors for downstream analysis, scHPF was first run with two more factors than the number of PhenoGraph clusters within the lineage, then subsequently increased nine times by steps of one, for a total of nine separate factorizations (that is, *k* = (17, 18, 19 … 250). To achieve consistent granularity across lineages, we chose the factorization in which ~90% of the variance in a cells’ expression (on average) was explained by the top 7 factors, given by 22 factors for endothelial and fibroblasts lineages and 27 factors for myeloid lineage cells.

After matrix factorization in human samples, we identified gene programs associated with T_reg_ cell presence in LuAd tumors by calculating the Spearman correlation between the log_2_ average factor cell score and log_2_ T_reg_ cell proportion of CD45^+^ cells in each sample. This calculation was also performed using the T_reg_ cell proportion of CD3^+^ cells in each sample to ensure consistency; however, the T_reg_ cell proportion of CD45^+^ cells are referenced in the primary results (Extended Data Fig. 9a). We assessed the stability of gene programs using a similar strategy to that used for mouse above, and robustness of factor associations to T_reg_ cell presence was assessed by the same correlation calculation using matched factors in a separate run of scHPF factorized using a different value of *k*. The relationship of factors across lineages was assessed by the pairwise Spearman correlation of log_2_ average factor cell scores in each sample from one factor to all other factors. Only samples with enough representative cells were used for correlation analysis in each lineage (>5 cells in endothelial and fibroblast, >20 cells in myeloid). Sample 16 was removed from all factor correlations due to outlier values driven by high IFN signatures, and sample 17 was removed from endothelial correlations due to outlier values driven by low cell numbers.

To identify conserved gene programs (factors) in endothelial, fibroblast and myeloid cells between human and mouse tumors, we compared the gene scores of orthologous genes. First, the genes used for factorization were filtered for orthologs that had a one-to-one correspondence between species (Ensembl 85 annotations) and were expressed in both species. A gene was assigned to a factor if its gene score was two standard deviations greater than the mean of gene scores for all genes in that factor. Then, a Jaccard similarity score was calculated between all mouse and human factors of a given lineage by dividing the number of shared assigned genes by the number of unique assigned genes in each pair of factors. The *z*-score of Jaccard values for all human factors against each mouse factor was used to identify human factors with greater homology to a mouse factor than background. Typically, a Jaccard similarity score greater than 0.06 in the endothelial lineage and 0.07 in the fibroblast and myeloid lineages and would define one (and no more than three) factors in human with homology to a mouse factor.

The validity of factor mappings across species was assessed by examining the genes shared between conserved factors to ensure they belonged to coherent biological programs (inflammation, angiogenesis, and so on). To find genes with similarly high scores across conserved factors, we normalized the gene score for each gene by the sum of its scores across all factors (fraction of total gene score), which also enabled comparison across factorizations. This was used to compare T_reg_ cell-associated inflammation and hypoxia programs in Fig. 4g; we compared normalized gene scores from the sum of human factors 4 and 5 to those in mouse factor 15, as these corresponded to the same underlying biological process across species (see below). In Fig. 4g, genes were listed as conserved if they were assigned (as described above) to both the human and mouse factors being compared. VEGF-regulated genes in endothelial cells were identified using gene sets derived by Dhainaut et al.^31^ with data from the CytoSig database, which houses public cytokine response datasets for many cell types and treatment pairs (https://cytosig.ccr.cancer.gov/).

In certain cases, gene or cell scores for several factors were summed to relate an underlying biological process to similar gene expression programs in mouse (as above) or T_reg_ cell proportion across human participants. An underlying biological process (for example, inflammation) could be split across several factors due to similar but nonoverlapping expression programs (for example, cell-type-specific signaling) or very similar expression programs with sample-specific or condition-specific effects. Comparisons including only partial signal in these cases, when only a single factor was compared to another entity, could mask associations to the broader biological program. In Fig. 4f, we summed cell loadings for human endothelial factors 3, 4 and 5 to relate the conserved T_reg_ cell-responsive endothelial expression program to T_reg_ cell proportion across tumor samples. We reasoned that these factors were related to a shared underlying biological process because they were each individually negatively associated with T_reg_ cell proportion across samples to various degrees (Extended Data Fig. 9c), and their genes aligned with different components of T_reg_ cell depletion-induced expression program in mouse tumors: factor 3, aCap; factor 4/5, inflammation and hypoxia with features of the mouse activated VEC (Fig. 4e). Additionally, factors 4 and 5 shared inflammation-relevant genes (*IL6*, *CSF3*) but with different sample specificities, which indicated that sample-specific effects rather than the underlying biology could have separated this gene program across two human factors (Extended Data Fig. 9d). Therefore, a summed factor score was found to be more appropriate in capturing certain endothelial gene program relationships to T_reg_ cell proportion.

### Expression heat maps

Once we identified the factors of interest in each of the cell types, based on our definition of higher average cell score in DT compared to control conditions, we zoomed into the genes that contributed the most to those factors. We were particularly interested in understanding the genes that drive the factor score in a specific subpopulation of cells. In our analysis, we sought to focus on specific subtypes with the highest average cell score for the factor. As such, we isolated the cell types of interest and correlated the factor usage (cell scores) with gene expression. Details of the subsetting are provided in Supplementary Table 19. To elaborate, we provide an example: we identified factors 9, 14 and 22 to be enriched in DT-treated cells compared to control in the fibroblast subpopulation in the mouse tumor model. These factors had the highest cell usage scores among the COL14A1 subtype. Therefore, to identify genes that are driving these factors and ensure that we focus on gene programs specific to the COL14A1 subtype, we subset this cell type of interest and correlate factor usage with gene expression. In cases where the cell type of interest was small (for example, the inflammatory capillary subset in endothelial cells in the mouse tumor model), we subsetted the cell type of interest combined with the phenotypically most similar cell type (for example, we grouped the inflammatory capillary subset with aCap in the mouse tumor model endothelial cells). This ensured we had sufficient cell numbers to compute the correlation and allowed us to identify genes specific to the cell type of interest in contrast to its nearest phenotypically similar subtype.

To this end, we correlated gene expression against the cell scores in the isolated set of cells and identified the top 200 most correlated genes as being relevant to that factor for that specific subpopulation. To ensure that the correlation scores were not influenced by any potential outliers (cells with deviant cell scores), we compared our results against correlation computed between the imputed gene expression and imputed factor cell scores (using MAGIC^57^, nPCs = 20, *k* = 30, *k*_a_ = 10, *t* = 4). In both scenarios, we obtained highly similar results. The expression heat maps (Figs. 2i and 3i and Extended Data Figs. 5a,c, 6b and 7a,d) display the result from imputed data.

We followed the same procedure for the bleomycin injury model data.

### Spatial transcriptomics

#### Read mapping and quantification

We processed Visium ST data with the SpaceRanger pipeline from 10X Genomics (v1.3.1). The mkfastq function was used to generate FASTQ files from raw base calls and the count function was used in combination with a matched brightfield H&E-stained image to align to a modified mm10 genome, perform tissue detection and count UMIs for each spot. The modified genome consisted of Ensembl 100 annotations with an added transcript to detect DTR-GFP expressed from the *Foxp3* promoter (sDTR-eGFP). UMI counts were summed by gene symbol and sDTR-eGFP reads were summed together with Foxp3. All analyses of differential cell-type abundance or gene expression were performed in the first serial section of each biological sample to preserve the independence of observations. Gene expression counts were log normalized using SCTransform^67^ with Seurat (v4.1.1)^68^ to compare between spots. Spots with fewer than 1,000 UMIs were excluded from analysis.

#### Deconvolution of Visium spots to cell-type RNA fractions

Visium captures transcripts from sectioned tissue placed over 55-µm-diameter spots, such that each spot sums gene expression from multiple cells. We used the BayesPrism algorithm^25,26^ to deconvolve cell types present in each spot and thereby improve the effective resolution of the technology. As input, BayesPrism accepts a spot-by-gene count matrix and a scRNA-seq reference dataset labeled by cell type; it utilizes a Bayesian approach to jointly model cell-type fractions and cell-type-specific gene expression within each Visium spot.

In our ST analysis, we used two separate scRNA-seq references for deconvolution—one containing the small number of tumor cells (*N* = 239) captured in our study (‘non-merged reference’) and another containing cells from a separate study that sampled more tumor cells (*N* = 18,083) from specific tumor sub-states (‘merged reference’, detailed below). The merged reference was used to assess the presence of granular transcriptional states within tumors, while the non-merged reference was used to study accessory cell populations without the influence of batch effects (data from two separate studies) or confounding during deconvolution (limited resolution between normal epithelial and certain tumor states, that is, AT2 versus AT2-like tumors). The non-merged reference includes scRNA-seq data solely consisting of cells from identical experimental conditions to the ST data (KP tumor-bearing lungs treated with PBS or DT; data from Fig. 2) and was used for all analyses in Fig. 4 and Extended Data Fig. 7c,e,f. Cell fraction estimates from the non-merged reference were used to distinguish tumor spots from normal spots because of the better-matched experimental characteristics, the capture of tumor cells from the T_reg_ cell-depleted state and the lower chance of similarity to normal EC types by using all tumor cells as a single reference population. The cell-type fraction estimates of tumor sub-states from the merged reference were used for analysis in Fig. 5 and Extended Data Fig. 8 only in tumor spots defined using the non-merged reference. Additional details of scRNA-seq reference construction and applications of the cell-type fraction estimates are mentioned below.

#### Selection of cell types and marker genes

The accuracy and reliability of cell fraction estimates depends on the presence of features in Visium data that are specific to labeled populations (highly specific cell-type markers give better deconvolution), the transcriptional distance between populations (better separated populations give better deconvolution) and how closely matched the scRNA-seq reference is with populations profiled in situ by ST. We thus optimized both gene selection and cell-type label granularity in our scRNA-seq reference and leveraged the ability of BayesPrism to encode separate cell states within a population to better match the reference in specific conditions (that is, control versus T_reg_ cell depleted).

Feature selection before deconvolution can improve the signal-to-noise ratio by removing genes that are irrelevant to cell type but behave similarly to relevant genes, and it can also mitigate the influence of genes that change due to batch effects. We therefore chose to focus on cell-type marker genes in our deconvolution, which is a recommended option in BayesPrism. Marker genes were computed by conducting pairwise *t*-tests across cell types (findMarker function in SCRAN) using log-normalized data. We defined marker genes by a minimum *P* value of 0.05 and minimum log fold-change value of 0.25 across all comparisons. Genes with fewer than ten counts across all Visium sections, or those detected in fewer than five cells in scRNA-seq data, were removed in addition to ribosomal genes, mitochondrial genes and genes associated with the cell cycle (https://github.com/dpeerlab/spectra/).

We merged highly similar cell types to avoid confounding deconvolution. To ensure adequate resolution between cell types, we computed marker genes as described above, starting at the most granular level of annotation and iteratively merging cell populations with their closest neighbor (by transcriptional distance), until each cell population had at least 30 marker genes. This included collapsing Artery/Vein with gCap cells (labeled as gCap); CD8^+^ T cells, effector T cells, exhausted CD8^+^ T cells, MAIT, gdT, T_H_2, naïve T cell, activated T cell, T_reg_ and ILC2 populations (T cell/ILC2); B and plasma cells (B cells); monocyte and Csf3r^+^ monocytes (monocyte); cDC1 and cDC2 (cDC); and Csf3r^+^ neutrophil, Ccl3^+^ neutrophil, and Siglecf^+^ neutrophil (neutrophil). We further merged the inflammatory capillary population with aCap cells (aCap), and Arg1^+^ with C1q^+^ macrophage populations (macrophages), as these are arguably specialized cell states of the same overarching cell type. Cycling T cells were also removed to prevent misassignment to tissue regions with increased expression of cell cycle-related genes. The resulting filtered scRNA-seq reference comprised 4,219 marker genes and 23,178 cells labeled as 26 cell populations (see Extended Data Fig. 7a for full list of cell populations included).

While our feature selection strategy ensured adequate resolution between cell types, transcriptional heterogeneity within cell types can also influence deconvolution. BayesPrism initially performs inference at the cell-state level, which can account for condition-specific heterogeneity in transcriptional states within cell types during deconvolution. Cell states can be captured by the algorithm through cell-type-specific expression estimates but can also be included as labels in the reference data. We observed substantial transcriptional shifts in accessory cell populations between control and T_reg_ cell-depleted conditions by scRNA-seq (Fig. 2 and Extended Data Figs. 4 and 5), and thus labeled cells from these accessory populations to help capture heterogeneity within cell types. Control and T_reg_ cell-depleted states were assigned for aCap, gCap, LECs, Col13a1^+^ and Col14a1^+^ fibroblasts, pericytes, myofibroblasts, AT1, AT2, cDC, macrophage, alveolar macrophage, neutrophil and monocyte populations in the scRNA-seq reference. BayesPrism sums cell-state fractions at the cell-type level before the final update step and downstream analysis.

Following cell-state definition, BayesPrism was run jointly on serial sections, to allow sharing of information across more spots during the final update step and filtering out of genes whose expression fraction (reads/total reads) was greater than 0.01 in 10% of Visium spots. For the robustness and reproducibility analysis, each section was deconvolved independently.

KP tumor cells are known to adopt a range of recurrent cell states as they progress^27–30^. To assess tumor transcriptional states and their relation to T_reg_ cell depletion, we performed a second deconvolution using BayesPrism across Visium spots with a scRNA-seq reference containing more granular tumor-state labels. Given the limited number of tumor cells in our reference (239 cells), we decided to incorporate scRNA-seq data from Yang et al.^28^, which contains ~50,000 KP tumor cells (referred to as KP-Tracer data), to more accurately assign general states within tumor regions. A key advantage of BayesPrism is that it can incorporate single-cell data from multiple sources, which do not need to be matched with our data. The algorithm uses cell types in the scRNA-seq reference as a prior for possible cell states in the Visium data, while disregarding cell-type fractions in the reference.

To minimize computational burden and sample-specific biases, we processed the KP-Tracer data by removing mutation-specific and mesenchymal cell states (these were largely sample-specific), and downsampling the remaining tumor states to a maximum of 2,000 cells (for balanced sampling), leaving 18,083 cells. The original tumor-state labels of EMT-1, EMT-2 and pre-EMT were combined (labeled as EMT), as were early gastric, late gastric and gastric-like populations (gastric) to limit deconvolution to the most representative overarching tumor states. These cells were combined with our accessory cell data in the same count matrix, with tumor cells removed. Marker gene selection and gene filtering were performed as above but adding 24 genes upregulated in AT2 cells relative to the AT2-like tumor state (*t*-test on log-normalized scRNA-seq data with adjusted *P* value < 0.001, log fold change > 1, expressed in 15% more cells relative to AT2-like) to better discriminate tumor from normal states. The final merged reference contained 40,787 cells and 4,546 genes after cell and feature selection, and we ran BayesPrism deconvolution with it using identical settings to the non-merged reference.

Visium data are very noisy. To discriminate robust evidence for cell type, we only included cell-type fractions above background at particular spots, using the same mixed-model strategy as the compute.background function from SpaceFold^26^, with modifications detailed below. Specifically, for each cell type in each tissue section, a gamma mixture model with two components was fit for cell-type fraction (gammamixEM from mixtools^69^), and a Gaussian mixture model with two components was fit for the summed deconvolved gene expression values (Mclust from Mclust^70^) across all spots. Mixture model distributions were checked for agreement with data structure by histogram and overlay of the fitted distribution. After determining parameters for the mixture components, we identified spots with >70% posterior probability of being assigned to the mixture component having the higher mean and used the minimum value of these as a threshold for calling a cell type ‘present’. Cell-type thresholds below 0.001 were reset to 0.001, and summed deconvolved gene expression thresholds below 50 were reset to 50. To enable comparison across tissue sections and prevent erroneous cutoffs due to tissue-specific composition, the median of cell fraction and summed deconvolved gene expression cutoffs across all eight tissue sections was used for each cell type. These values were subsequently refined with the guidance of H&E staining (see below for details). Illustrations of spot binarization for the presence of specific cell types are shown in Fig. 4a and Extended Data Fig. 7d,e.

#### Assessing robustness and accuracy

We assessed the robustness and accuracy of cell-type RNA fraction estimates before proceeding further with analysis downstream of our deconvolution. We performed bootstrap analysis to determine robustness to the sparse capture of Visium. Specifically, we ran BayesPrism on one tissue section with each spot randomly downsampled to 90% of its reads, repeated this 20 times, and calculated the Spearman correlation of cell-type fraction estimates between the original and each downsampled deconvolution. Cell fraction estimates across spots were highly consistent, with Spearman correlations ≥ 0.87 for all trials (Extended Data Fig. 7a). We next compared average cell-type fraction between individually deconvolved serial sections across all samples, validating the expectation that cell-type fractions captured by serial sections are highly similar (Spearman *R* = 0.99; Extended Data Fig. 7b). To ensure consistency between our two deconvolution approaches, we compared cell-type fractions of non-tumor accessory cells with and without additional tumor states from the KP-Tracer study in our scRNA-seq reference. Average log(cell-type RNA fraction) values from each tissue section were highly correlated (Spearman *R* = 0.97), suggesting that deconvolved accessory populations were generally not influenced by tumor RNA, and that the inclusion of tumor cells from a separate study did not impact accessory cell deconvolution (Extended Data Fig. 7c). One exception was in resolving AT1-like and AT2-like epithelial states, which are highly similar to several of the added tumor states; the added states likely improved their resolution in tumor regions, but not in non-tumor regions, due to transcriptional similarity with normal epithelial states.

Cell-type assignment was cross-referenced with the underlying tissue histology from matched H&E-stained brightfield images to confirm accurate positioning of cell types where possible (Extended Data Fig. 7d,e). For example, spots deemed to possess different capillary types, pericytes and alveolar macrophages were consistent with the literature and anatomical features (Extended Data Fig. 7d). gCap and artery/vein cells were localized around blood vessels and alveoli, with some penetration into tumor areas, whereas aCap cells were mainly distributed over alveoli and surrounding tumor areas, consistent with their propensity to surround areas of injury^48^. Pericytes were localized around blood vessels and bronchi, consistent with published annotations^60^, and alveolar macrophages were concentrated in areas surrounding tumor regions, as previously shown^45^. LECs, DCs and B cells are all expected in areas containing lymphoid aggregates emanating from a lymphatic vessel and were indeed detected in these regions by our ST analysis (Extended Data Fig. 7e). Moreover, regions representing part of an IC signaling niche were found to have higher neutrophil cell-type fraction (Fig. 4f), which was readily apparent in the aligned tissue section due to the unique appearance of neutrophils in H&E staining (Fig. 5f).

Upon assessing marker gene expression and inspecting histology, we noted that several cell types including AT2, gCap, MSCs, monocytes and LECs had more modes in the distribution of their cell-type fractions and summed deconvolved gene expression values across spots, likely due to regional variation in cell-type composition and read density. To account for this variation, we reset the presence/absence thresholds for these cell types as above but using mixture models with three mixture components instead of two. As a result, the minimum value from spots assigned to the mixture component with the second highest mean (one above background mixture component) with >70 posterior probability was used as a threshold. The median cell-type fraction and summed deconvolved gene expression threshold values of the three-component mixture models across all tissue sections was applied to all spots (as for two-component models above).

#### Analysis of gene program usage across conditions

To assess the differential use of gene programs between control and T_reg_ cell-depleted conditions identified by factor analysis in scRNA-seq data, we used the AddModuleScore function in Seurat to compute the relative log-normalized expression of each factor’s genes relative to a random set of background genes with similar average expression in the tissue. Specifically, all genes were split into 24 expression bins and 100 control features were randomly selected for each feature in the input gene program from a corresponding bin. The average log-normalized expression of control features was then subtracted from the average log-normalized expression of the features of interest to derive a module score. Module scores were computed across spots from all four samples at the same time. A *t*-test was performed to compare gene program module scores in control and T_reg_ cell-depleted conditions for gene programs of interest, and *P* values were adjusted by Benjamini–Hochberg correction. To measure the difference in relevant cellular contexts, comparisons were restricted to spots with cell-type fractions above background for cell types in which the gene program of interest was found to be differential by scRNA-seq, creating a table comparing cell type by gene program of interest across conditions (Fig. 4b). The visualization of specific module scores was performed in Figs. 4c,d and 5g.

#### Definition of signaling niches

Certain gene programs that increased their abundance in both our scRNA-seq and ST analysis following T_reg_ cell depletion shared many genes across endothelial, fibroblast and myeloid lineages. This included factors that contained many IFN-stimulated genes (IFN factors) and factors that contained genes related to IC and hypoxia signaling (IC factors). To determine shared genes between IFN and IC factors, genes were assigned to each relevant factor from the mouse scRNA-seq in the same way as detailed in ‘Comparison of human and mouse factors’ and the intersection of genes across all three lineages for IFN or IC factors was taken. The IFN factors were defined as fibroblast factor 9, endothelial factor 19 and myeloid factor 17. IC factors were defined as fibroblast factor 22, endothelial factor 15 and myeloid factor 21. The module score of shared genes for IFN (*N* = 103 genes) or IC (*N* = 18 genes)-related gene programs (See Supplementary Table 12 for gene lists) was then used to define ‘signaling niches’ or Visium spots where a common signaling pathway may drive downstream gene expression in several colocalized cell types.

To assign spots to a signaling niche, we took advantage of the fact that most spots across all tissue sections did not show signal for IFN or IC gene programs. Therefore, we modeled the background rate of these gene programs by fitting their module scores plus a pseudocount of one to a gamma distribution using maximum likelihood estimation (fitdistr from MASS package^71^) across all spots on the four biologically independent sections being analyzed. Alignment with the gamma distribution was checked by a histogram of the gene scores and density overlay of the fit distribution. The module score corresponding to an upper tail probability of 0.01 in the fit distributions was then used as a threshold above which spots were assigned to that signaling niche. Assignment of spots to the IFN or IC signaling niche was not mutually exclusive and gave 397 spots assigned to IC niches, 330 spots assigned to IFN niches and 21 spots assigned to both. An illustration of spot assignment to signaling niches is shown in Fig. 4e.

#### Cell-type enrichment in signaling niches

To assess the presence of different cell types within signaling niches relative to background cell-type fractions across the tissue (Fig. 4f), we took a random sample of 100 spots across all tissue sections and averaged the fraction of each cell type, then repeated for 10,000 iterations to form an empirical probability distribution of mean cell-type fractions of randomly selected spots. The empirical *P* value was calculated as the fraction of iterations in our empirical distribution with an average cell-type fraction above the average for all spots in a given signaling niche (IFN or IC). Empirical *P* values were adjusted by Benjamini–Hochberg correction to account for multiple hypothesis testing. To measure the magnitude of enrichment for each cell type, the log_2_ average cell-type fractions from the total empirical distribution were subtracted from average cell-type fraction values from either signaling niche.

#### Definition of tumor-state regions

To classify spots within tumor lesions into areas of consistent transcriptional phenotypic state (‘tumor lesion areas’), we first selected spots with tumor RNA above background (detailed above) and used cell-type fractions from the deconvolution with the merged reference. To visualize the co-occurrence of tumor states, we *z*-scored fractions of tumor states in tumor spots and hierarchically clustered the spots into seven groups (R cutree with *k* = 7) using Pearson correlation distance and average agglomeration (Extended Data Fig. 8a). This analysis revealed that tumor spots were typically dominated by a single tumor state. When plotted in their tissue context, we found that they often aggregated spatially (Fig. 5a), providing further support for the presence of consistent transcriptional phenotypes within lesional areas.

Each of the seven clusters was then labeled based on the tumor state with the highest cell-type fraction in the cluster. The validity of cluster labels was assessed by the expression of tumor-state marker genes defined by previous studies^28^. We found clearly higher expression of marker genes in their corresponding cluster relative to other tumor clusters (Extended Data Fig. 8b). The classification of tumor spots in their tissue location is shown in Fig. 5b and Extended Data Fig. 8c. In H&E staining, the location of different tumor states often corresponded to a noticeable change in histology, further supporting our classifications. For instance, neighboring gastric and high-plasticity regions also exhibited a more differentiated morphology in the gastric tumor area and less structure in the high-plasticity area (Fig. 5f,g). While our strategy increased the resolution of tumor transcriptional states in our deconvolution, there may be additional tumor states in situ that are not contained within our merged scRNA-seq reference due to heterogeneity of the model. In these cases, the cell-type fractions from missing tumor states would be assigned to the closest transcriptional neighbor.

Tumor lesion areas were defined by separating connected components (contiguous spots in tissue) of the same tumor-state cluster. AT1-like and AT2-like tumor-state clusters were merged for tumor lesion area definition because of the higher degree of mixing between these tumor states observed previously^29^ and in our analysis (Extended Data Fig. 8a). Only lesion areas greater than six spots were kept for subsequent analysis, to avoid micrometastases or regions dominated by tumor edges due to sectioning. This resulted in 47 and 38 tumor lesion areas in control and T_reg_ cell-depleted tissue sections, respectively. Tumor lesion areas were deemed to have an immune response in T_reg_ cell-depleted tissue sections if >10% of constituent spots were part of an IC or IFN signaling niche (Fig. 5e).

### Differential expression of tumor areas

We were interested in detecting differential gene expression between tumor lesion areas. We first collected all spots in tumor lesion areas (1) exhibiting an immune response and (2) exhibiting no response after T_reg_ cell depletion (defined in the paragraph above), then performed a Wilcoxon rank-sum test between the two groups of spots. SCTransform log-normalized values were used as input and only genes detected in at least 10% of spots in either condition and with an average log fold-change value > 0.25 between conditions were tested. We detected 259 genes that were differentially higher in responding tumors and 142 genes that were higher in non-responding tumors at Benjamini–Hochberg-adjusted *P* value < 0.01 and log fold-change > 0.5 (Fig. 5d and Supplementary Table 15). The SCTransform log-normalized expression levels of specific genes associated with non-responsiveness to T_reg_ cell depletion are shown in Fig. 5e.

### Statistics

For all mouse experiments, statistical analyses were performed using GraphPad Prism 9 and are detailed in the figure legends. Mice were allocated randomly to experimental groups. No statistical methods were used to predetermine sample sizes but our sample sizes are similar to those reported in previous publications^5,13^. Data collection and analysis were not performed blind to the conditions of the experiments.

Statistical tests used for analysis of RNA-seq and ST data are described. For scRNA-seq and ST, count data were assumed to be distributed according to a negative binomial distribution and log-transformed data according to a normal distribution. In other analyses, data distribution was assumed to be normal but this was not formally tested.

scRNA-seq data analysis was performed using custom code relying primarily on Python v3.8.11 using Scanpy v1.8.1 package for basic pre-processing and analysis. Visualization of the data was done using MulticoreTSNE v0.1 implementation of *t*-SNE in Python, and clustering was done using PhenoGraph v1.5.7 package in Python. Factor analysis was done using scHPF v0.5.0 implementation in Python v3.7.11. Differential abundance testing between scRNA-seq conditions was performed using Milo v1.3.4. Identification of factors (Hungarian matching algorithm) was implemented using the linear_sum_assignment module in optimize submodule of SciPy (v1.7.1) in Python (v3.8). For human factor analysis, Spearman correlation coefficients and *P* values were calculated in R using ggpubr (0.4.0) and results were visualized using ggplot2 v3.3.5.

### Reporting summary

Further information on research design is available in the Nature Portfolio Reporting Summary linked to this article.

## Online content

Any methods, additional references, Nature Portfolio reporting summaries, source data, extended data, supplementary information, acknowledgements, peer review information; details of author contributions and competing interests; and statements of data and code availability are available at 10.1038/s41590-023-01504-2.

## Supplementary information

Supplementary Information Supplementary Fig. 1 and Supplementary Materials and Methods.

Reporting Summary

Supplementary Table 1 Tumor_48_gene_counts

Supplementary Table 2 KP_factors

Supplementary Table 3 KP_Endo 3,8,15,17_factors_on_subsets

Supplementary Table 4 KP_Fib_9,14,22_factors_on_subsets

Supplementary Table 5 KP_Mye 5,15,17,21,23_factors_on_subsets

Supplementary Table 6 KP_Significant_factors_on_cell_subsets

Supplementary Table 7 Bleomycin_factors

Supplementary_Table 8 Bleomycin_Endo_12,13,15_factors_on_subsets

Supplementary Table 9 Shared_activated_VEC_KP_3_bleomycin_15 genes

Supplementary Table 10 Shared_Mac_KP_23_bleomycin_0_genes

Supplementary Table 11 Cell_populations_in_situ

Supplementary Table 12 IC_IFN_shared_genes

Supplementary Table 13 Scrna_tumor_de_mast

Supplementary Table 14 Scrna_tumor_de_fgsea

Supplementary Table 15 Ic_de_wilcox

Supplementary Table 16 Table_S16_Sample composition

Supplementary Table 17 Tumor_sample_info

Supplementary Table 18 LuAd_factors_(all lineages)

Supplementary Table 19 Glossary_of_Treg_compensatory_factors

Supplementary Table 20 Antibodies

Source Data Supplementary Fig. 1 Factor robustness.

## Data Availability

Raw and processed bulk, scRNA-seq and Visium data from mouse are available from the Gene Expression Omnibus under super series accession GSE202159. Human tumor scRNA-seq data are available at the Human Tumor Atlas Network (HTAN) data coordinating center web platform (https://humantumoratlas.org/). Source data are provided with this paper.

## Source data

Source Data Fig. 1 Statistical source data.
Source Data Fig. 2 Statistical source data.
Source Data Fig. 3 Statistical source data.
Source Data Fig. 4 Statistical source data.
Source Data Figs. 5 and 6 Statistical source data.
Source Data Fig. 7 Statistical source data.
Source Data Fig. 8 Statistical source data.
Source Data Extended Data Fig. 1 Statistical source data.
Source Data Extended Data Fig. 2 Statistical source data.
Source Data Extended Data Fig. 3 Statistical source data.
Source Data Extended Data Fig. 4 Statistical source data.
Source Data Extended Data Fig. 5 Statistical source data.
Source Data Extended Data Fig. 6 Statistical source data.
Source Data Extended Data Fig. 7 Statistical source data.
Source Data Extended Data Fig. 8 Statistical source data.
Source Data Extended Data Fig. 9 Statistical source data.
Source Data Extended Data Fig. 10 Statistical source data.

## Code availability

No new algorithms were developed for this paper. All analysis code is available at https://github.com/dpeerlab/Treg_depletion_reproducibility/.
