# Supplementary material for: Conserved transcriptional connectivity of regulatory T cells in the tumor microenvironment informs new combination cancer therapy strategies
Source: Nat Immunol. 2023 May 1;24(6):1020–35. doi: 10.1038/s41590-023-01504-2 (PMC10232368; doi:10.1038/s41590-023-01504-2)
Supplement: Supplementary file 1 — Supplementary Fig. 1 and Supplementary Materials and Methods. [file 41590_2023_1504_MOESM1_ESM.pdf]

nature immunology

Article https://doi.org/10.1038/s41590-023-01504-2

Conserved transcriptional connectivity of regulatory T cells in the tumor microenvironment informs new combination cancer therapy strategies

In the format provided by the authors and unedited

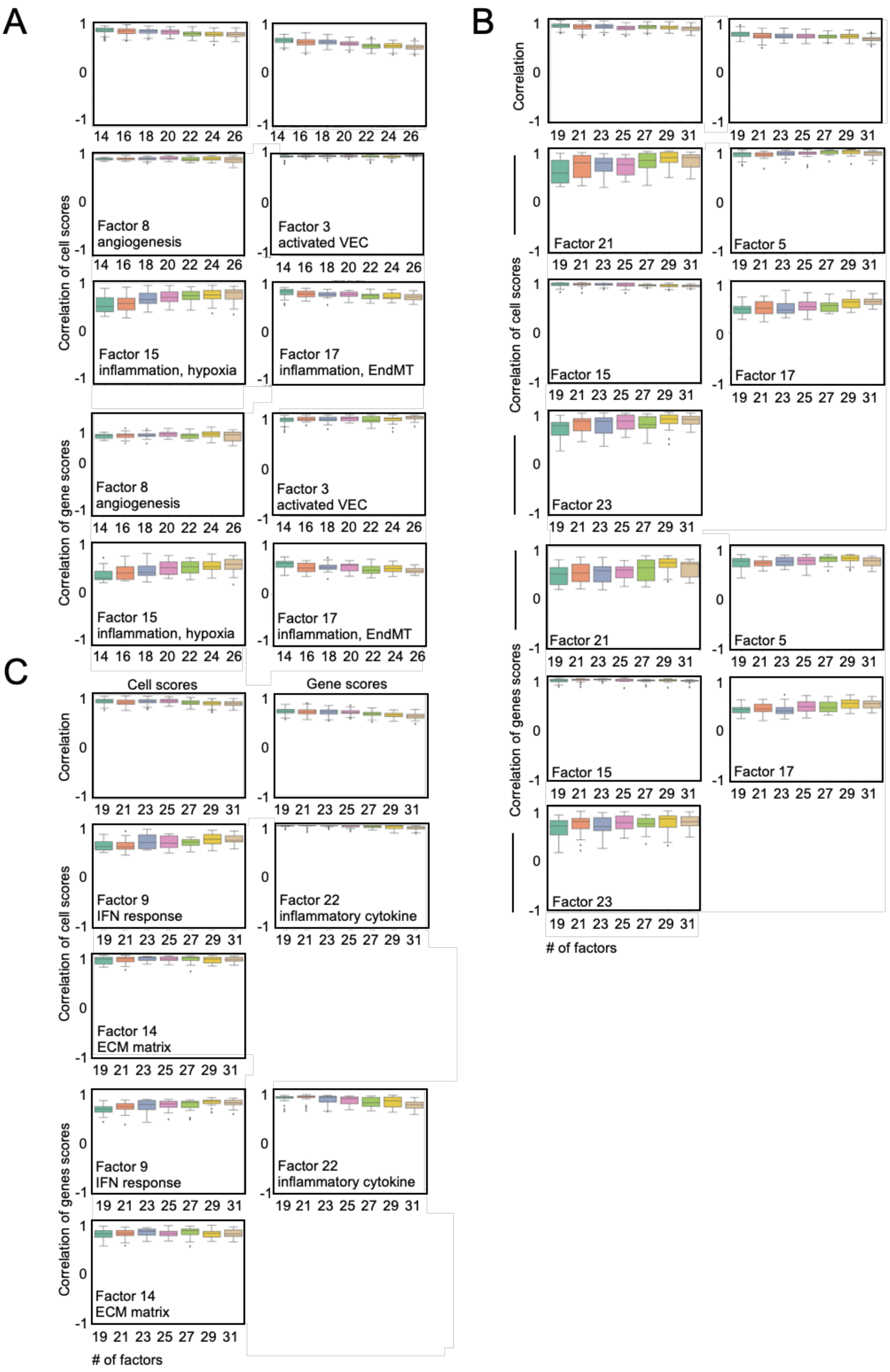
A
B
C
Correlation
Correlation of cell scores
Correlation of gene scores
Correlation of genes scores
Factor 8
angiogenesis
Factor 3
activated VEC
Factor 15
inflammation, hypoxia
Factor 17
inflammation, EndMT
Factor 21
Factor 5
Factor 23
Cell scores
Gene scores
Factor 9
IFN response
Factor 22
inflammatory cytokine
Factor 14
ECM matrix
# of factors

Supplementary Figure S1. Robustness of factor analysis of single cell transcriptomes of vascular endothelium, fibroblasts and macrophages in lung KP adenocarcinomas.

(A) Box plots illustrate robustness of the tumor model endothelial factors to random initializations (top). Median self-correlation of factors under random initializations (N=20 iterations) is shown. Box plots depict robustness of cell scores of specific factors of interest (as specified in each box plot) to changes in the number of factors (middle). The correlation of cell scores across 20 iterations for a range of number of factors is shown. Box plots depict robustness of gene scores of specific factors of interest (bottom). The box indicates the quartiles, with the median indicated by the horizontal line inside the box and the whiskers indicate 1.5 times the interquartile range (as implemented in seaborn.boxplot package in Python). (B) Same as (A) for fibroblast factors. (C) Same as (A) for myeloid factors.

Supplementary Materials and Methods

Computational analysis of scRNA-seq data

<u>*Basic pre-processing and lineage identification*</u>

Sequence reads from each sample were aligned to the mm10 mouse genome reference and processed with default parameters for the 10X single-cell 3' library using the SEQC pipeline[54]. SEQC performs multi-mapped read resolution and UMI correction to output a cell-by-gene count matrix for each sample, and it distinguishes cells from empty droplets, removes cells with high mitochondrial content (>20%) and removes cells that express few unique genes. The resulting molecule count matrix from each sample was concatenated, incorporating any gene expressed in at least one sample, to obtain the final raw molecule count matrix (Table S11). Similar cell-types from different samples overlapped in 2D visualizations, indicating an absence of strong batch effects in the combined samples (Extended Data Fig.2B, right). Genes expressed in fewer than 10 cells in the combined data were removed from further analysis. The library size (total RNA count in each cell) distribution was bimodal; we removed low-quality cells with library size below 500 to isolate the upper mode. For the bleomycin-induced lung injury model (injury), we removed cells with library size below 300 counts. After removing low-quality cells, we again discarded genes expressed in fewer than 10 cells. The data was then median library size normalized (i.e. each cell was divided by its total counts and multiplied by the median library size across all cells) followed by log (base 2) transformation with a pseudocount of 0.1. The resulting clean combined data consisted of 27606 cells and 16435 genes (KP) and 26556 cells and 17119 genes (injury).

<u>*Analysis of individual lineages (mouse samples)*</u>

Endothelial cells. Endothelial cells were selected from the combined data annotation and genes expressed in at least 10 endothelial cells were preserved for downstream analysis. The cells were then clustered using PhenoGraph (k = 30) on principal components (nPCs = 50). Doublets, clusters of cells with low library size, clusters of cells with high mitochondrial or ribosomal expression, or cells with library size less than 1000 were removed from further processing. This resulted in 2815 cells by 12131 gene matrix with a median library size of 3344 (KP) and 1351 cells and 11485 genes with a median library size of 4518 (injury). The cells were then re-clustered using Phenograph with Leiden community detection on top 50 principal components (k = 30) and visualized using tSNE (nPCs = 50, perplexity = 100). Obtained clusters were then annotated into respective cell-types using known markers (Extended Data Fig.3A).

Fibroblasts. We isolated cells identified as fibroblasts in the combined data and analyzed them separately. First, genes expressed in fewer than 10 fibroblasts were discarded from analysis. We then evaluated the library size distribution and removed cells with low library size (< 3.4 on $\log_{10}$ scale) and cells identified as doublets by *Scrublet*[61]. For injury, this resulted in only 339 cells, and as such we did not perform downstream analysis on fibroblasts. For KP, we then clustered the cells using Phenograph[19] with Leiden community detection[62] on Jaccard corrected knn graph (k = 30) on top of the principal components (nPCs = 50). One of the clusters was found to have high mitochondrial expression and was removed from analysis. We finally obtained a cell-by-gene matrix with 3791 cells, 13588 genes and a median library size (total counts) of 7343. The clusters were then annotated into specific cell-types based on the average expression of known markers that delineate subtypes of fibroblast cells (Extended Data Fig.3B).

Myeloid cells. Similarly, we isolated myeloid cells from the combined data and retained genes expressed in at least 10 cells for downstream analysis. The cells were clustered using Phenograph[18] with Leiden community detection[62] on the top 50 principal components (k = 30). Cells belonging to clusters with a high proportion of low library size cells or doublets (as identified by *Scrublet*[61]) or those with library size less than 2500 (KP) or 3000 (injury) molecules were discarded from downstream analysis. The final data consists of 4718 cells and 12786 genes with a median library size of 8589 (KP), and 8299 cells and 14523 genes with median library size of 11211 (injury). The cells were re-clustered using Phenograph with Leiden community detection (k = 30) on the top 50 principal components and visualized using tSNE (perplexity = 100). The obtained clusters were then annotated into specific cell-types using known marker expression (see Extended Data Fig.3C).

Tumor cells. Epithelial cells were isolated from the combined data and clustered using Phenograph with Leiden community detection on the top 50 principal components (k = 30). SEQC aligned reads were scanned for the presence of C>T mutations at position Chr6:145246770, representing instances of the *Kras*^{G12D} mutation. The proportion of cells with at least one read count for the G12D mutation was assessed for each Phenograph cluster. Two clusters were found to be highly enriched for the *Kras* mutation and had higher expression of *Kras* signaling associated genes including various *Mapk1*, *Map2k1*, *Map2k2*, *Nras*, and *Pik3ca*. Based on this evidence, these two epithelial cell clusters were annotated as tumor cells.

Cell-type annotation

For each lineage, we performed cell-type annotation by computing the average expression of known cell-type markers in each cluster. The results were then displayed as a heatmap and used to assign a cell-type label to each cluster. To ensure that the cell-type annotation was not impacted by sparsity of gene expression, we denoised and imputed gene expression in each of the lineages using MAGIC[56] with parameters (k = 30, ka = 10, t = 3). However, we observed equivalent results using imputed or un-imputed data.

LuAd associated cell lineage identification

5,000 highly variable genes (HVG) were selected using the variance of standardized feature values scaled by the expected variance derived from a loess curve fit to the relationship of the log transformed variance and mean across all genes[63]. Dimensionality reduction was performed by PCA using log-normalized expression of HVGs as input. To assign single cells to broad lineages, PhenoGraph clustering (k = 40) was performed using the top 50 PCs and a minimum cluster size of 100 cells. Cells that did not fit into a cluster were removed and remaining cells were assigned to lineages by marker gene expression detailed in previous studies (Fig. 4B and S9A)[58,64]. t-SNE was performed on the combined and log normalized data (perplexity = 30) for a visual representation of global clustering. Visualization of the t-SNE colored by sample showed sample specific epithelial populations but mixed immune, fibroblast, and endothelial populations (Extended Data Fig.9B). This is consistent with previous reports[53] and obviated the need for batch correction in single cell lineage specification.

Analysis of individual lineages

Dimensionality reduction and clustering was performed in a similar fashion within T/NK, Myeloid, Endothelial, and Fibroblast lineages with several modifications. Within lineages, log-normalized expression values were calculated by the SCRAN package[65] with default parameters. Clusters used for SCRAN normalization were the result of the quickCluster command from the SCRAN package for coarse cluster assignment. In all lineages further analyzed, genes were removed from subsequent clustering analysis if they were expressed in fewer than 0.1% of cells or 10 cells, or if they corresponded to mitochondrial transcripts, highly expressed ncRNAs or genes known to influence clustering (*NEAT1*, *MALAT1*, *TMSB4X*), ribosomal RNA or protein genes, immunoglobulin transcripts, hemoglobin genes, or T-cell receptor variable regions. Additionally, genes associated with cellular stress[66] were removed from the T/NK and Myeloid lineages as they were found to confound cell-typing of clusters.

T/NK cells. T/NK cells were isolated from the combined data based on lineage markers. After gene filtering there were 12,840 genes and 38,241 cells. PhenoGraph clustering with Leiden community detection (k = 40, minimum cluster size = 50) was then performed on the top 50 PCs calculated using 2,000 HVGs selected as previously described. t-SNE dimensionality reduction was then performed (perplexity = 30) for cluster visualization. After examining PhenoGraph clusters with low library size and high levels of putative ambient RNA, additional cells were removed if they had a library size below 1,000 UMIs and under 400 genes detected, leaving 36,783 cells, and then the cells were re-clustered using the same strategy. Treg cells were defined by a T/NK PhenoGraph cluster (cluster C1) with high expression of FOXP3, CD3E, CD4, and IL2RA and other Treg enriched genes (Fig. 4C, Extended Data Fig.9C). This cluster contained all the cells exhibiting canonical Treg gene expression and included cells from all samples (Extended Data Fig.11D), indicating additional batch correction was not necessary for Treg definition. Treg cell counts in each sample were divided by the total counts of hematopoietic cells (Myeloid, B, T/NK, Neutrophil lineages) in each sample to provide a normalized metric for use in relation to gene programs identified through factor analysis (Extended Data Fig.10A). Treg count divided by the number of cells in $CD3^{+}$ PhenoGraph clusters was strongly correlated with the Treg hematopoietic fraction across samples (Extended Data Fig.10B) which indicated robustness of this metric.

Endothelial cells. Endothelial cells were isolated from the combined data based on lineage markers. After gene filtering there were 13,459 genes and 2,299 cells. PhenoGraph clustering was performed (k = 30, minimum cluster size = 15) on the top 50 PCs calculated using 2,000 HVGs selected as previously described. A smaller k value relative to other lineages was used due to the smaller number of endothelial cells. t-SNE dimensionality reduction was then performed (perplexity = 30) for cluster visualization. PhenoGraph clusters with low library size and potentially high ambient RNA fractions were removed, leaving 2,272 cells for subsequent analysis.

Fibroblast. Fibroblast cells were isolated from the combined data based on lineage markers. After gene filtering there were 15,181 genes and 2,888 cells. PhenoGraph clustering was performed (k = 40, minimum cluster size = 50) on the top 50 PCs calculated using 2,000 HVGs selected as previously described. t-SNE dimensionality reduction was then performed (perplexity = 30) for cluster visualization. PhenoGraph clusters with low library size and potentially high ambient RNA fractions were removed, leaving 2,652 cells for subsequent analysis.

Myeloid cells. Myeloid cells (monocytes, macrophages, dendritic cells, mast cells, neutrophils) were isolated from the combined data based on lineage markers. After gene filtering there were 14,813 and 11,323 cells. PhenoGraph clustering with Leiden community detection (k = 40, minimum cluster size = 50) was then performed on the top 50 PCs calculated using 2,000 HVGs selected as previously described. t-SNE dimensionality reduction was then performed (perplexity = 30) for cluster visualization. After identifying cells with low library size and potentially high ambient RNA fractions, an additional clustering was performed with the same parameters on only monocytes, macrophages, and dendritic cells to further isolate problematic cells. Cells not forming a cluster in this subsequent round were removed, leaving 11,260 cells for subsequent analysis.

After global and lineage specific QC of Human scRNA-seq data 82,991 cells across all lineages remained. All lineages were re-clustered in the same manner they were originally after filtering out low quality cells.
