# Supplementary material for: Conserved transcriptional connectivity of regulatory T cells in the tumor microenvironment informs new combination cancer therapy strategies
Source: Nat Immunol. 2023 May 1;24(6):1020–35. doi: 10.1038/s41590-023-01504-2 (PMC10232368; doi:10.1038/s41590-023-01504-2)
Supplement: Supplementary file 2 — Reporting Summary [file 41590_2023_1504_MOESM2_ESM.pdf]

nature research

Corresponding author(s): Alexander Y Rudensky
Dana Pe'er

Last updated by author(s): Apr 1, 2023

Reporting Summary

Nature Research wishes to improve the reproducibility of the work that we publish. This form provides structure for consistency and transparency in reporting. For further information on Nature Research policies, see our Editorial Policies and the Editorial Policy Checklist.

Statistics

For all statistical analyses, confirm that the following items are present in the figure legend, table legend, main text, or Methods section.

n/a	Confirmed	
☐	☒	The exact sample size (*n*) for each experimental group/condition, given as a discrete number and unit of measurement
☐	☒	A statement on whether measurements were taken from distinct samples or whether the same sample was measured repeatedly
☐	☒	The statistical test(s) used AND whether they are one- or two-sided<br>*Only common tests should be described solely by name; describe more complex techniques in the Methods section.*
☒	☐	A description of all covariates tested
☐	☒	A description of any assumptions or corrections, such as tests of normality and adjustment for multiple comparisons
☐	☒	A full description of the statistical parameters including central tendency (e.g. means) or other basic estimates (e.g. regression coefficient) AND variation (e.g. standard deviation) or associated estimates of uncertainty (e.g. confidence intervals)
☐	☒	For null hypothesis testing, the test statistic (e.g. *F*, *t*, *r*) with confidence intervals, effect sizes, degrees of freedom and *P* value noted<br>*Give P values as exact values whenever suitable.*
☒	☐	For Bayesian analysis, information on the choice of priors and Markov chain Monte Carlo settings
☒	☐	For hierarchical and complex designs, identification of the appropriate level for tests and full reporting of outcomes
☒	☐	Estimates of effect sizes (e.g. Cohen's *d*, Pearson's *r*), indicating how they were calculated

Our web collection on statistics for biologists contains articles on many of the points above.

Software and code

Policy information about availability of computer code

Data collection	Flow cytometry data were collected on an LSRII using FACS Diva v8.0 (BD), or on Aurora using SpectroFlo v2.2.0.3 (Cytek)<br>Bulk RNA-seq libraries were sequenced on HiSeq (Illumina).<br>scRNA-seq libraries were sequenced on NovaSeq (Illimina).
Data analysis	Flow cytometry data were analyzed using FlowJo v 10.6.1 (BD)<br>GraphPad Prism v9 was used for statistical analysis<br>All Images were processed and analyzed using ImageJ package v2.0.0-rc-69/1.52p<br>For bulk RNA sequencing STAR aligner v2.7.3a was used for alignment. R v3.6.0 was used for generating count matrices and DESeq2 was used for Principal Component Analysis, to identify differentially expressed genes and for Spearman correlations calculations and for hierarchical clustering and generation of K-means heatmaps.<br>scRNA-seq data analysis was performed using custom code relying primarily in Python v3.8.11 using Scanpy v1.8.1 package for basic pre-processing and analysis. Visualization of the data was done using MulticoreTSNE v0.1 implementation of tSNE in Python and clustering was done using PhenoGraph v1.5.7 package in Python. Factor analysis was done using scHPF v0.5.0 implementation in Python v3.7.11.<br>Differential abundance testing between scRNA-seq conditions was performed using Milo v1.3.4.<br>Identification of factors (Hungarian matching algorithm) was implemented using the linear_sum_assignment module in optimize submodule of scipy package (v1.7.1) in Python (v3.8).<br>For human factor analysis, Spearman correlation coefficients and p values were calculated in R using ggpubr (0.4.0) and results were visualized using ggplot2 v3.3.5.

For manuscripts utilizing custom algorithms or software that are central to the research but not yet described in published literature, software must be made available to editors and reviewers. We strongly encourage code deposition in a community repository (e.g. GitHub). See the Nature Research guidelines for submitting code & software for further information.

Data

Policy information about availability of data

All manuscripts must include a data availability statement. This statement should provide the following information, where applicable:

- Accession codes, unique identifiers, or web links for publicly available datasets
- A list of figures that have associated raw data
- A description of any restrictions on data availability

Raw and processed bulk, single cell RNA-seq, and Visium data from mouse are available from Gene Expression Omnibus (GEO) at super series accession GSE202159. Human tumor scRNA-seq data is available at the Human Tumor Atlas Network (HTAN) data coordinating center web platform (data.humantumoratlas.org).

Field-specific reporting

Please select the one below that is the best fit for your research. If you are not sure, read the appropriate sections before making your selection.

☒ Life sciences ☐ Behavioural & social sciences ☐ Ecological, evolutionary & environmental sciences

For a reference copy of the document with all sections, see nature.com/documents/nr-reporting-summary-flat.pdf

Life sciences study design

All studies must disclose on these points even when the disclosure is negative.

Sample size	No statistical methods were used to pre-determine sample sizes but our sample sizes are similar to those reported in previous publications 5,13. Human sample size was based on the largest amount of patient samples available from the HTAN study collection that were primary LuAD or local met.
Data exclusions	In human factor analysis only samples with sufficient cell numbers (Fibroblast > 5, Endothelial > 5, Myeloid > 20) in a given lineage were used for association of factor usage to Treg proportion. Additionally, one sample with high IFN activation and another with low cell numbers and inconsistent factor estimates within the endothelial lineage were removed from the same analysis.
Replication	All experiments in the study were performed at least twice with consistent results except for single cell sequencing and spatial transcriptomics, where no repetition is customary in the field 54, 72.
Randomization	Mice were sex and age matched. Mice were allocated randomly to experimental groups. Only continuous trends between cell proportion and factor use were assessed across all patients and therefore controls based on sample groupings are not relevant.
Blinding	Data collection and analysis were not performed blind to the conditions of the experiments. It is customary in the field that no blinding is needed5,13.

Reporting for specific materials, systems and methods

We require information from authors about some types of materials, experimental systems and methods used in many studies. Here, indicate whether each material, system or method listed is relevant to your study. If you are not sure if a list item applies to your research, read the appropriate section before selecting a response.

Materials & experimental systems

n/a	Involved in the study	
☐	☒	Antibodies
☒	☐	Eukaryotic cell lines
☒	☐	Palaeontology and archaeology
☐	☒	Animals and other organisms
☐	☒	Human research participants
☒	☐	Clinical data
☒	☐	Dual use research of concern

Methods

n/a	Involved in the study	
☒	☐	ChIP-seq
☐	☒	Flow cytometry
☒	☐	MRI-based neuroimaging

Antibodies

Antibodies used	For immunofourecence, the following antibody conjugates were used:<br>Ab supplier cat #<br>GFP-AF488 ThermoFisher A12311<br>CD3-AF532 ThermoFisher 58-0032-82<br>CD4-AF647 BioLegend 100530<br>TCRβ-PE ThermoFisher 12-5961-83

GP38-PE-Cy7 BioLegend 127412
Lyve-1-APC R&D Systems FAB2125A
CD11c AF594 BioLegend 117346
F4/80-AF647 Tonbo Bioscience #20-4801-U100

For flow cytometry, the following antibody conjugates were used:
Ab supplier cat #
CD45-BV510 BioLegend 103137
CD45-BV570 BioLegend 103136
TCRβ-PE-eFluor610 ThermoFisher 61-5961-82
CD3-PerCP-Cy5.5 Tonbo Bioscience 65-0031-U100
CD3-BV650 BioLegend 100229
CD4-PerCP-Cy5.5 Tonbo Bioscience 65-0042-U100
CD4-ef450, ThermoFisher 48-0042-82
CD4-BV510 BioLegend 100553
CD8a-BV605 BioLegend 100744
CD8a-PE-e610 ThermoFisher 61-0081-82
CD8-BV711 BioLegend 100759
CD31-PE BioLegend 102508
EPCAM-AF647 BioLegend 118212
GP38-PE-Cy7 BioLegend 127412
CD11b-BV605 BioLegend 101257
CD11b-BV480 BD Biosciences 566117
CD11c-APC BD Biosciences 550261
CD11c-BV605 BioLegend 117334
GR1-FITC, ThermoFisher 11-5931-82
GR1-AF532 ThermoFisher 58-5931-82
MHCII-redfluor710 Tonbo Bioscience 80-5321-U100
TER-119-FITC, ThermoFisher 11-5921-82
B220-BUV496 BD Biosciences 564662
F4/80-APC Tonbo Bioscience 20-4801-U100
CD44-ef450 ThermoFisher 48-0441-82
CD62L-BV605 BioLegend 104438
KI67-AF700 BioLegend 652420
IFNg-ef450 Tonbo Bioscience 755-7311-U100
TNFa-BV605 BioLegend 506329
NK1.1-APC-eF780 ThermoFisher 47-5941-82
CD64-APC BioLegend 139306
Ly6G-PE-Cy7 BioLegend 127618
CD19-PE-Cy5 BioLegend 115510
Ly6C-BV711 BioLegend 128037
Siglec-F-BV421 BD Biosciences 562681
CCR8-AF-647 BioLegend 150303
CCR8-PE R&D FAB8324P025

For in- vivo experiments, the following antibodies where used
Ctrl IgG BioXcell BE0130
anti CCR8 BioLegend 150302
anti VEGF R&D AF-493-M

Validation

All above antibodies were well validated commercial clones or preps rutinely QC'ed by the manufacturer. Please refer to the spec sheets on the respective vendors' website for technical information and detail by searching the catalog numbers provided.

Animals and other organisms

Policy information about studies involving animals; ARRIVE guidelines recommended for reporting animal research

Laboratory animals

Animals were housed at the Memorial Sloan Kettering Cancer Center (MSKCC) animal facility under specific pathogen free (SPF) conditions according to institutional guidelines. Mice used in this study had no previous history of experimentation or exposure to drugs. Adult male and female mice (6 weeks or older) were used for all experiments.
genetic strains used were Foxp3GFP-DTR and KrasLSL-G12D Trp53fl/fl. 10,13.

Wild animals

No wild animals were used in this study.

Field-collected samples

No field collected samples were used in this study.

Ethics oversight

All studies were performed under protocol 08-10-023 and approved by the MSKCC Institutional Animal Care and Use Committee

Note that full information on the approval of the study protocol must also be provided in the manuscript.

Human research participants

Policy information about studies involving human research participants

Population characteristics	Metadata for patient tumor samples including race, gender, and other characteristics can be found in Supplementary Table 17.
Recruitment	Patients with lung adenocarcinoma undergoing a surgical resection or tissue biopsy at Memorial Sloan Kettering Cancer Center (MSKCC) were identified and biospecimens collected prospectively from 2017 to 2020. All patients from whom biospecimens were obtained provided informed consent for an MSKCC-wide biospecimen collection and analysis protocol. Recruitment was designed to capture a wide, unbiased swath of heterogeneous disease, with a slight emphasis on EGFR-mutated tumors with a high propensity to transform to more aggressive subtypes. Biases may be present related to this recruitment design, the race, sex, smoking status and the general patient population of MSKCC.
Ethics oversight	Use of all patient material and data described in this manuscript was performed under ethical approval obtained from the Memorial Sloan Kettering Cancer Center Institutional Review Board (Study numbers: 06-107 and 12-245).

Note that full information on the approval of the study protocol must also be provided in the manuscript.

Flow Cytometry

Plots

Confirm that:

☒ The axis labels state the marker and fluorochrome used (e.g. CD4-FITC).

☒ The axis scales are clearly visible. Include numbers along axes only for bottom left plot of group (a 'group' is an analysis of identical markers).

☒ All plots are contour plots with outliers or pseudocolor plots.

☒ A numerical value for number of cells or percentage (with statistics) is provided.

Methodology

Sample preparation	For isolation of immune and stromal cells, lungs were perfused, placed into 5 ml Eppendorf tubes containing 400[illegible]l of cold serum-free RPMI and chopped with scissors (1-2 mm). Lung fragments were placed in 2-3 ml of pre-warmed digestion medium (RPMI 1640, 10mM HEPES buffer, 1% penicillin–streptomycin, 1% L-glutamine, liberase (Sigma-Aldrich #05401020001) and 1U/ml DNase I (Sigma-Aldrich #10104159001)) (2-3 ml) and incubated for 30 min at 37°C. After digestion supernatant was collected and cells were resuspended in ice-cold RPMI1640 containing 5% FCS (ThermoFisher #35010CV), 1mM HEPES (Corning #MT25060CI), 1% penicillin–streptomycin (Corning #MT30002CI) and 200mM L-glutamine (Corning #MT25005CI). After additional digestion for 1 hr of the remaining tissue, both digested cell fractions passed through a 100um strainer (Corning #07-201-432), washed and FACS sorted. For cell isolation from transplanted. KP tumor-bearing mice, tumors were placed into 5 ml Eppendorf tubes containing 400ul of cold serum-free RPMI1640, chopped with scissors, and incubated in digestion medium containing 1 mg/ml collagenase (Sigma #11088793001) and 1U/ml DNase-I (Sigma-Aldrich #10104159001) and beads on a shaker at 37°C for 1 hr. For cytokine production measurements, cells were incubated at 37°C, 5% CO2 for 3hr in the presence of 50ng/ml phorbol-12-myristate-13-acetate (Sigma-Aldrich #P8139), 500ng/ml ionomycin (Sigma-Aldrich #I0634), 1ug/ml brefeldin A (Sigma-Aldrich #B6542) and 2µM monensin (Sigma-Aldrich #M5273). Cells were stained with Ghost Dye Red 780 (Tonbo #13-0865) or Zombie NIR Flexible Viability Kit (BioLegend #423106) and a mixture of fluorophore-conjugated antibodies for 30 min at 4oC cells, washed and fixed with 1% PFA (Electron Microscopy Sciences #15710). For intracellular staining, cells were fixed and permeabilized with the BD Cytofix/Cytoperm Kit or with the Thermo Fisher Transcription Factor Fix/Perm Kit according to manufacturers' instructions and analyzed on a BD LSR II flow cytometer or sorted on a BD Aria II flow cytometer. Post-sort cell purity was routinely higher than 95%.
Instrument	Samples were analysed on BD LSR II, Aurora (Cytek), or sorted on a BD Aria II.
Software	Flow cytometry data were collected on an LSR II using Diva v8.0 (BD) or Aurora using SpectroFlo v2.2.0.3 (cytec) and analyzed using FlowJo v 10.6.1 (BD)
Cell population abundance	Cells were sorted with <95% purity.
Gating strategy	All cells were gated based on FSC-A and SSC-A to exclude debris. Then doublets were excluded by plotting FSC-H vs FSC-W, followed by SSC-H vs SSC-W. Then dead cells were excluded based on live/dead dye negative. CD45 positive and negative cells were gated next. All further gating strategies are illustrated in the supplementary material.

☒ Tick this box to confirm that a figure exemplifying the gating strategy is provided in the Supplementary Information.
